# Bimetallic Au–Pd nanoparticles supported on silica with a tunable core@shell structure: enhanced catalytic activity of Pd(core)–Au(shell) over Au(core)–Pd(shell)†

**DOI:** 10.1039/d1na00489a

**Published:** 2021-08-09

**Authors:** Gauravjyoti D. Kalita, Podma P. Sarmah, Golap Kalita, Pankaj Das

**Affiliations:** Department of Chemistry, Dibrugarh University Dibrugarh Assam 786004 India pankajdas@dibru.ac.in; Department of Physical Science and Engineering, Nagoya Institute of Technology (NiTech) Nagoya Aichi Japan-466-8555

## Abstract

A facile ligand-assisted approach of synthesizing bimetallic Au–Pd nanoparticles supported on silica with a tunable core@shell structure is presented. Maneuvering the addition sequence of metal salts, both Au_core_–Pd_shell_ (Au@Pd–SiO_2_) and Pd_core_–Au_shell_ (Pd@Au–SiO_2_) nanoparticles were synthesized. The structures and compositions of the core–shell materials were confirmed by probe-corrected HRTEM, TEM-EDX mapping, EDS line scanning, XPS, PXRD, BET, FE-SEM-EDX and ICP analysis. The synergistic potentials of the core–shell materials were evaluated for two important reactions *viz.* hydrogenation of nitroarenes to anilines and hydration of nitriles to amides. In fact, in both the reactions, the Au–Pd materials exhibited superior performance over monometallic Au or Pd counterparts. Notably, among the two bimetallic materials, the one with Pd_core_–Au_shell_ structure displayed superior activity over the Au_core_–Pd_shell_ structure which could be attributed to the higher stability and uniform Au–Pd bimetallic interfaces in the former compared to the latter. Apart from enhanced synergism, high chemoselectivity in hydrogenation, wide functional group tolerance, high recyclability, *etc.* are other advantages of our system. A kinetic study has also been performed for the nitrile hydration reaction which demonstrates first order kinetics. Evaluation of rate constants along with a brief investigation on the Hammett parameters has also been presented.

## Abstract 2

A facile approach for the synthesis of bimetallic Au–Pd core–shell nanoparticles with tunable core and shell atoms and their synergistic influences on chemoselective hydrogenation of nitroarenes to anilines and hydration of nitriles to amides.

## Graphical abstract

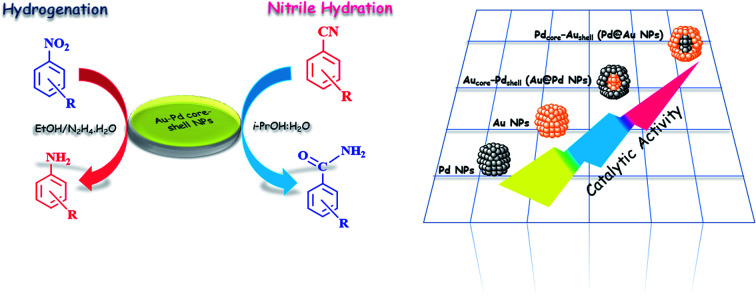

## Introduction

The concept of mutualism centers around the idea of integrating two metals in perfect correlation with each other, thereby exhibiting distinct superiority in chemical and physical properties over their monometallic counterparts.^1^ In the past few years, bimetallic nanostructured materials have attracted enormous attention because of their multifunctional applications in diverse areas such as catalysis,^2,3^ materials sciences (in optoelectronic and magnetic devices),^4^ medical biotechnology,^5^*etc.* Among various bimetallic nanoparticles, the Au–Pd system has been one of the most studied systems because of its applicability in a number of catalytic reactions such as alcohol oxidation,^6^ CO oxidation,^7^ dehydration of formic acid,^8^ dye degradation,^9^ hydrodechlorination,^10^ chemoselective hydrogenation^11^ and so forth. However, the activity and selectivity of such bimetallic catalysts not only depend on metal systems alone but also on the structural motif or morphology of such materials. Thus, virtual control over material structures is one of the most desirable, yet challenging, areas in nanoparticle research. Among various nanostructured materials (*e.g.* alloy, core–shell, heterostructure, *etc.*), special attention has been paid to core–shell structures for their unprecedented success in the field of catalysis.^12,13^ In fact, there are multiple instances where core–shell Au–Pd nanoparticles exhibited improved catalytic efficacies compared to their alloy counterparts.^14,15^

It is worth noting that the catalytic properties of a core–shell material vary not only with the size and morphology but also with the ordering of core and shell metals. Although, there have been multiple reports on the synthesis of core–shell Au–Pd NPs,^16–19^ the ability to control the order of core and shell metals is still contemplated as one of the most difficult tasks in materials research. Incidentally, the majority of the core–shell Au–Pd systems reported till date are typically galvanic in nature, where the Au atom being more noble prefers to occupy the core and the Pd atom being less noble forms the shell. On the contrary, anti-galvanic core–shell nanoparticles with Pd_core_–Au_shell_ structures are relatively few and limited in scope.^20,21^ In fact, a number of theoretical studies involving molecular dynamics simulation^22,23^ and density functional theory (DFT)^24^ have envisaged that in a core–shell structure, the Pd_core_–Au_shell_ arrangement is deemed more stable than the Au_core_–Pd_shell_ arrangement. This theoretical outcome is further reinforced by a few experimental studies that demonstrated the synthesis of stable Au–Pd core–shell nanoparticles with Pd_core_–Au_shell_ arrangement and their application as catalysts for different organic transformations like hydrogenation,^20^ selective oxidation of alcohol,^21^ photocatalytic oxidation,^25^*etc.*

Inquisitively, a majority of the Au–Pd core–shell catalysts reported to date are colloidal in nature, while from the catalytic perspective, supported NPs are more demanding as they facilitate catalyst separation, recovery and subsequent recycling. However, unlike colloidal nanoparticles, precise architectural control in a supported system is complicated as such systems predominantly produce Au_core_–Pd_shell_ structures *via* a galvanic replacement pathway,^26–28^ though from the catalytic perspective, nanoparticles with Pd_core_–Au_shell_ structures could be more intriguing. Thus, it is of great interest to rationally design supported Au–Pd core–shell nanoparticles with tunable core and shell atoms and to explore their catalytic potentials. Several experimental methods are currently employed for the synthesis of such core–shell systems *viz.* the radiolytic method,^29^ photocatalytic approach,^30^ vapor deposition method,^20^ sequential reduction approach,^31^ surfactant-mediated method,^21^ dendrimer-assisted method,^31^ ligand-based stabilization^32^ method, *etc.* Among these, the seed-mediated or sequential reduction approach that involved reduction of a shell metal onto a preformed metallic core was found to be the most attractive as it usually provided better control over the tunable core–shell property,^33^ thereby controlling the activity and selectivity. It has been noted that in the seed-mediated process, the nature of the surface capping ligand often plays the most crucial role in controlling the particle morphology. Thus, a judicious choice of the capping ligand also plays a significant role^34^ in this respect. Recently, we have reported the synthesis and catalytic activities of monometallic Pd-nanoparticles supported on silica stabilized by a phosphine ligand.^35^ In this work, we have extended the scope of this system to a bimetallic platform by using a ligand stabilized less noble metal as the core which is coated with a more noble metal counterpart acting as the shell. The order is reversed through coordination of the same ligand with the more noble metal followed by coating with the less noble metal. Thus, by controlling the addition sequence of the metal salts, core–shell Au–Pd nanoparticles with Au_core_–Pd_shell_ and Pd_core_–Au_shell_ arrangements can be easily accessed. The synergistic potentials of the core–shell materials were explored for two important reactions *viz*. hydrogenation of nitroarenes to amines and hydration of nitriles to amides.

## Experimental

### Materials

2-Diphenylphosphinoethyl-functionalized silica gel (0.7 mmol g^−1^ loading; 200–400 US mesh), HAuCl_4_ and PdCl_2_ were purchased from Sigma-Aldrich. All solvents, substrates and other chemicals were purchased from Acros Organics (purity > 98%), TCI chemicals and Merck (purity > 99%).

### Characterization

X-ray diffraction patterns were acquired on a Rigaku Ultima IV Powder X-ray diffractometer using Cu-K_∞_ as the source (*λ* = 1.5406 Å) with a scan rate of 2*θ* = 4° min^−1^ over a scanning angle of 5–80°. X-ray photoelectron spectroscopy (XPS) analysis was carried out using an FEI PHI 5000 Versa Prob II system. Surface area measurements were carried out at liquid nitrogen temperature on a Quantachrome Autosorb-iQ 2010 analyzer (ASIQA) using N_2_ gas as the adsorbent. Surface area calculations are done from the isotherm using the Brunauer–Emmett–Teller (BET) model and pore size distributions were calculated using the Barrett–Joyner–Halenda (BJH) model. Samples were degassed at 100 °C for 2 h before performing the experiments. A JEOL-JEM2010+ electron microscope (HRTEM) with an accelerating voltage of 200 keV was used to obtain high resolution transmission electron microscopy (HRTEM) images of the materials. Line scanning spectra were obtained using a special specimen stage atomic resolution scanning transmission electron microscope, JEOL-ARM200F equipped with an aberration corrector on the incident probe, which provides a nominal resolution of 0.1 nm at an acceptance semi angle of 26.5 mrad. A JEOL-JEM2010+ electron microscope (HRTEM) with an accelerating voltage of 200 keV was used to obtain high resolution transmission electron microscopy (HRTEM) images of the materials. A ZEISS SIGMA field emission scanning electron microscope (FESEM) with a lanthanum hexaboride (LaB_6_) filament and tungsten field emission source (FES) was used to obtain high resolution field emission microscopy (FESEM) images of the materials. The machine operates at an acceleration voltage on the order of magnitude of 0.5 to 30 kV and extreme vacuum of ∼10–6 Pa in the column of the microscope. The metal contents in the nanomaterials were measured using an ACROS, simultaneous inductively coupled plasma (ICP) spectrometer equipped with an R.F. generator (1.6 kW, 27.12 MHz), at wavelengths in the range of 130–770 nm. Catalytic hydrogenation reactions were carried out under appropriate reaction conditions in a sealed Teflon coated glass tube mounted on an aluminum block synthesizer slot with an LED sensor (model: PPS-5511, dimensions: 465W, 335L, 295H, Make: Eyela, USA). Catalytic nitrile hydration reactions were performed accordingly under specific reaction conditions. Gas chromatographic analysis of the reactants and products was performed on a 7820A Agilent GC system equipped with a capillary column (L 30 m, I.D. 0.25 mm), fitted with an FID and with *n*-dodecane as the internal standard. The mass spectroscopic analysis was performed using an MS-5965 Agilent analyzer.

### Synthesis of monometallic Au nanoparticles (SiO_2_@Au)

2.0 g of 2-diphenylphosphinoethyl functionalized silica gel was weighed and taken in a 100 ml round bottom flask containing 20 ml distilled water and stirred for 30 minutes and sonicated at 50 °C for 15 min to achieve complete dispersion. To the resultant mixture, 20 ml of 0.01 mmol HAuCl_4_ solution was added and stirred for an hour. The color of the silica gel changed to canary yellow. To the resultant mixture, 4 ml (51.2% v/v) of hydrazine solution was added dropwise over a period of 15 minutes and the color instantly changed to bright green. The stirring was continued for 1 h to ensure complete reduction of HAuCl_4_. Subsequently, following the previous experiment, the resultant solid mass was washed multiple times, dried at room temperature and kept in a desiccator. The vacuum dried material was named SiO_2_@Au.

### Synthesis of monometallic Pd nanoparticles (SiO_2_@Pd)

2.0 g of 2-diphenylphosphinoethyl functionalized silica gel was weighed and taken in a 100 ml round bottom flask containing 20 ml distilled water and stirred for 30 minutes and sonicated at 50 °C for 15 min to achieve complete dispersion. To the resultant mixture, 20 ml of 0.01 mmol Na_2_PdCl_4_ solution was added and stirred for an hour. The color of the silica gel changed to bright yellow. To the resultant mixture, 4 ml (51.2% v/v) of hydrazine solution was added dropwise over a period of 15 minutes and the color instantly changed to light brown. The stirring was continued for 1 h to ensure complete reduction of Na_2_PdCl_4_. Subsequently, this resultant solid mass was centrifuged and the residual filtrate was decanted. This process was performed thrice and the recovered solid mass was washed several times with small aliquots of distilled water, dried at room temperature and kept in a vacuum desiccator. The vacuum dried material was named SiO_2_@Pd.

### Synthesis of bimetallic Au–Pd nanoparticles with Au_core_–Pd_shell_ (Au@Pd–SiO_2_)

2.0 g of SiO_2_@Au was taken in a 100 ml round bottom flask containing 20 ml distilled methanol and stirred for 30 minutes to achieve complete dispersion. To the resultant suspension, 20 ml, 0.01 mmol Na_2_PdCl_4_ solution was added simultaneously and stirred for an hour. The color of the silica gel changed to pale yellow. To it 4 ml (51.2% v/v) hydrazine solution was added dropwise over a period of 15 minutes (rate = 0.26 ml min^−1^) and the color instantly changed to grey. The stirring was continued for another 1 h to ensure complete reduction of NaPdCl_4_. The resultant solid mass was filtered and washed several times with small aliquots of distilled water, dried at room temperature and stored in vacuum desiccators. The vacuum dried material was named Au@Pd–SiO_2_.

### Synthesis of bimetallic Au–Pd nanoparticles with Pd_core_–Au_shell_ (Pd@Au–SiO_2_)

2.0 g of SiO_2_@Pd was taken in a 100 ml round bottom flask containing 20 ml distilled methanol and stirred for 30 minutes to achieve complete dispersion. To the resultant suspension, 20 ml, 0.01 mmol HAuCl_4_ solution was added simultaneously and stirred for an hour. The color of the silica gel changed to bright yellow. To it 4 ml (51.2% v/v) hydrazine solution was added dropwise over a period of 15 minutes (rate = 0.26 ml min^−1^) and the color instantly changed to light brown. The stirring was continued for another hour to ensure complete reduction of HAuCl_4_. The resultant solid mass was filtered and washed several times with small aliquots of distilled water, dried at room temperature and stored in vacuum desiccators. The vacuum dried material was later named Pd@Au–SiO_2_.

### General procedure for hydrogenation

Hydrogenation of nitroarenes to corresponding amines was carried out by first charging 2 mmol of the substrate in 4 ml of the solvent taken in a Teflon coated glass tube mounted on a synthesizer slot. Initially 17.5 mg (0.07 mol%, 0.0014 mmol) of catalyst was weighed and subsequently added to the above reaction mixture. Thereafter, the synthesizer was heated to 80 °C with constant stirring (500 rpm) for the desired period. Once the reaction reached completion, the synthesizer was cooled to room temperature. The reaction mixture was separated from the catalyst by syringe filtration and analyzed using a GC-MS. The separated catalyst was washed repeatedly with ^*i*^PrOH and acetone, dried, and used further for recyclability tests.

### General procedure for nitrile hydration

The catalytic hydration of nitriles to corresponding amides was carried out by similarly charging 0.5 mmol of the desired substrate in 4 ml of the solvent taken in a Teflon coated glass tube mounted on a synthesizer slot. Instantly 12.5 mg (0.2 mol%, 0.001 mmol) of catalyst was added to the reaction mixture and the synthesizer was heated to 50 °C with constant stirring (400 rpm) for the desired period. After completion of the reaction, the synthesizer was cooled to room temperature. The reaction mixture was carefully separated from the catalyst, filtered and analyzed using a GC-MS. The separated catalyst was recovered, washed repeatedly, dried, and used further for recyclability tests.

### General procedure for heterogeneity tests

The reaction was performed with the desired substrate, solvent and the catalyst for a period of 15 minutes. The catalyst was carefully separated from the reaction mixture and the filtrate was later analyzed using a GC-MS. Subsequently, the solid mass was separated from the solution by filtration under hot conditions and the resultant filtrate was subjected to stirring for another hour under the same reaction conditions as eventually no increase in conversion was observed.

### General procedure for recyclability tests

For recycling experiments, after each run, the catalyst was washed several times with suitable solvents, dried to remove any occluded reactants and products physically attached to the surface of the catalytic material and reused for successive runs. Subsequently, the recovered catalyst was oven-dried at 95–100 °C for an hour and transferred to a reaction vessel containing the desired substrate and solvent, and reusability experiments were performed under identical conditions. Likewise, all the recyclability tests were performed maintaining the same stoichiometric ratios.

## Results and discussion

### Synthesis and characterization of silica-supported bimetallic Au–Pd nanoparticles

**Scheme 1:**
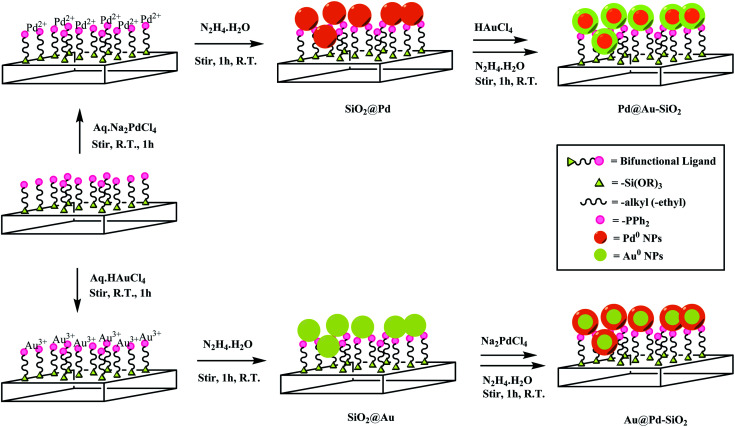
Synthesis of Au–Pd core–shell nanoparticles supported on phosphine-functionalized silica gel.

A schematic representation of the synthesis of silica-supported core–shell Au–Pd NPs with Au_core_–Pd_shell_ (Au@Pd–SiO_2_) and Pd_core_–Au_shell_ (Pd@Au–SiO_2_) has been shown in Scheme 1. A ligand-assisted sequential reduction approach has been followed. In the first step, silica-supported monometallic Pd NPs (SiO_2_@Pd) and Au NPs (SiO_2_@Au) were synthesized by reacting commercially available phosphine-functionalized silica gel with corresponding metal salts in the presence of hydrazine hydrate as the reducing agent following a previously reported protocol.^35^ The morphological properties and other characterization details of the monometallic materials have been presented in the ESI section (Fig. 4, 5, S1 and S2†). The HRTEM micrographs of SiO_2_@Au and SiO_2_@Pd (Fig. S1b and d†) demonstrated the formation of monodisperse Au and Pd NPs of uniform size and near-spherical shape homogeneously distributed throughout the silica matrix. The size-distribution histograms corresponding to the TEM micrographs indicated a diameter of 4.5 nm for Au NPs and 5.12 nm for Pd NPs (Fig. S1a and c† inset) respectively. In the second step, these monometallic Pd or Au NPs were employed as seeds and subsequent coatings with Au and Pd shells were made by treating methanolic suspensions of the preformed monometallic cores with an aqueous solution of the corresponding metal salts followed by reduction with hydrazine hydrate. It may be mentioned that this type of surface-ligand modulated seed-growth method has been recently reported by Kluenker *et al.* as an excellent method of synthesizing bimetallic Au–Pd nanoparticles with tuned morphology ranging from alloy to core–shell structures.^34^ Similarly, Esparza and co-workers synthesized core–shell nanoparticles using a similar technique and studied the development of Pd shells over Au cores using the Volmer–Weber model.^36^

**Fig. 1:**
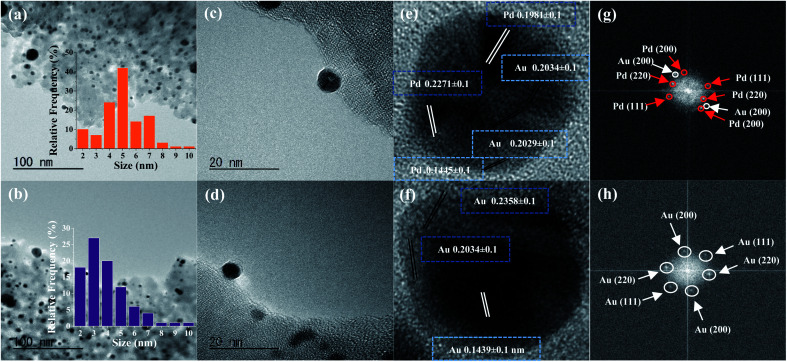
(a) TEM images and corresponding size distribution (inset, based on particle count 100) of Au@Pd–SiO_2_ and (b) Pd@Au–SiO_2_; (c) HRTEM image of Au@Pd–SiO_2_ and (d) Pd@Au–SiO_2_; high-magnification HRTEM image of a single particle depicting cross-sectional lattice planes and *d*-spacings of (e) Au@Pd–SiO_2_ and (f) Pd@Au–SiO_2_; fast Fourier transform (FFT) of Au, Pd and/or Au–Pd mixed reflections for (g) Au@Pd–SiO_2_ and (h) Pd@Au–SiO_2_.

The representative TEM micrographs of the bimetallic materials Au@Pd–SiO_2_ (Fig. 1a) and Pd@Au–SiO_2_ (Fig. 1b) indicated the formation of spherical nanoparticles. The size distribution histograms enumerate that the majority of the particles exhibited an average diameter of 5.42 nm for Au@Pd–SiO_2_ (Fig. 1a, inset) and 3.78 nm (Fig. 1b, inset) for Pd@Au–SiO_2_ respectively. The HRTEM micrographs of Au@Pd–SiO_2_ and Pd@Au–SiO_2_ exhibited nearly spherical morphology with slight inhomogeneity in size and differences in contrasts (Fig. 1c and d respectively)^37–39^ extending throughout the lattices.

The high-magnification TEM images for single particles of Au@Pd–SiO_2_ (Fig. 1e) and Pd@Au–SiO_2_ (Fig. 1f) showed the presence of distorted lattices^37^ indicating the presence of an interface between two different chemical compositions in the materials.

**Fig. 2:**
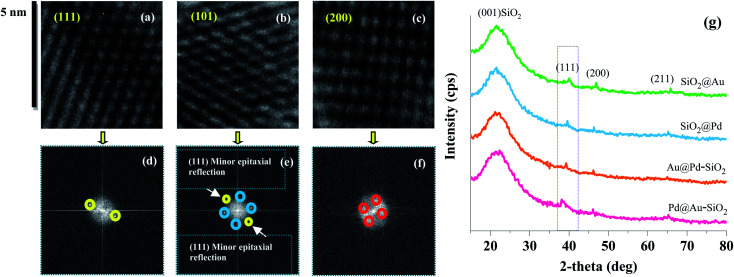
Crystalline lattice planes (a)–(c) and corresponding FFT maps (d)–(f) of Pd@Au–SiO_2_, (g) powder-XRD patterns of the nanoparticle incorporated silica-based materials: Pd@Au–SiO_2_, Au@Pd–SiO_2_, SiO_2_@Pd and SiO_2_@Au.

Usually, for a bimetallic system, the nature of crystal structure and *d*-spacing values tell whether a system adopts a core–shell or alloy structure.^40,41^ The fast Fourier transform (FFT) patterns presented in Fig. 1g and h reveal a face-centred cubic (FCC) arrangement for Au@Pd–SiO_2_ and Pd@Au–SiO_2_ materials indicating core–shell structures for both the materials.^40^ In Au@Pd–SiO_2_, the spots identified with white circles exhibit *d*-spacing values of 0.2029 ± 0.1 and 0.2034 ± 0.1 that can be attributed to the (200) lattice plane of Au (JCPDF 04-0784).^42^ Similarly, from the spots marked with red circles, *d*-spacings of 0.2271 ± 0.1, 0.1981 ± 0.1 and 0.1445 ± 0.1 were observed, which correspond to the (111), (200) and (211) planes for Pd (JCPDF 05-0681).^16^ Conversely, in the case of Pd@Au–SiO_2_, the spots recognized by white circles exhibited *d*-spacing values of 0.2358 ± 0.1, 0.2034 ± 0.1 and 0.1439 ± 0.1 that were consistent with (111), (200) and (220) lattice planes of Au (JCPDF 04-0784).^42^ In fact, the presence of peaks corresponding to the (111), (200) and (220) planes of Au and Pd was also observed from PXRD analysis of the Au–Pd materials (Fig. 2g). However, the peaks due to (200) and (220) planes observed at 2*θ* = 47.11°, 65.65° and 46.87°, 65.26° for Pd@Au–SiO_2_ and Au@Pd–SiO_2_ respectively were less intense compared to the (111) planes observed at 2*θ* = 39.05° and 38.11°. It may be noted that the peaks assigned to (111), (200) and (220) planes for Au and Pd crystallize in the fcc structure. Consequently, their respective peaks corresponding to these diffraction planes overlap.^43^ Apart from the crystal structure and *d*-spacing, lattice-mismatch is also an important parameter to identify the heteroepitaxial growth in a core–shell material. A large lattice-mismatch observed in a HRTEM usually leads to a non-coherent interface or a polycrystalline shell layer.^44^ In this respect, the FFT pattern corresponding to a HRTEM micrograph provides a better idea as multiple secondary reflections in the pattern usually act as clear evidence of potential lattice mismatches in the crystal structure.

In fact, the high-magnification HRTEM micrograph of Au@Pd–SiO_2_ (Fig. 1e) corroborated this and exhibited large lattice mismatches between the (111), (200) and (220) planes of Au and Pd indicative of a polycrystalline morphology. This is confirmed from the FFT pattern which depicts multiple secondary reflections for both Au and Pd. On the other hand, the high magnification HRTEM images of Pd@Au–SiO_2_ displayed a well-segregated core–shell interface with a mononuclear Pd core within an Au shell. As evidenced by the FFT pattern, a uniform heteroepitaxial growth with minimal lattice mismatch was observed between the (111), (200) and (220) planes corresponding to Au and Pd.

**Fig. 3:**
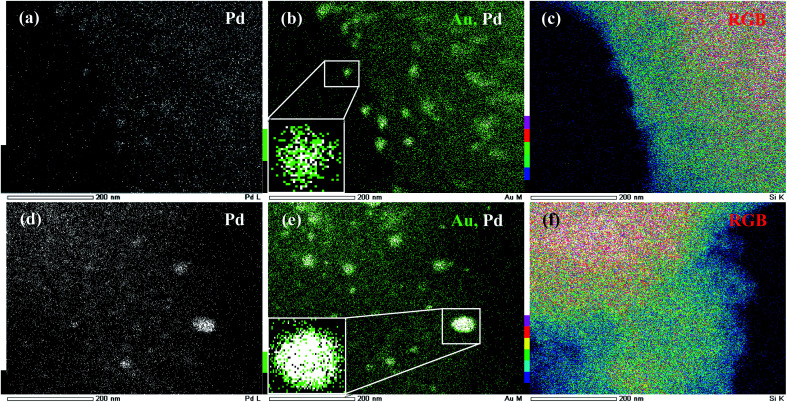
TEM-EDX mapping of Au@Pd–SiO_2_; (a) Pd map (white dots), (b) Au map (green dots) and Pd map (white dots) with high intensity of Au atoms at the core (inset magnified) and (c) RGB-overlay; TEM-EDX mapping of Pd@Au–SiO_2_; (d) Pd map (white dots), (e) Au map (green dots) and Pd map (white dots) with high intensity of Pd atoms at the core (inset magnified) and (f) RGB-overlay.

This is well confirmed by the pristine (no secondary reflections) FFT pattern (Fig. 1f and h) indicative of the typical monocrystalline nature of Pd@Au–SiO_2_ (Fig. 1g).^44^ Other than a minor epitaxial reflection of the (111) crystal plane upon the (101) plane (Fig. 2e) the crystal planes were free of any stack (Fig. 2d and f), fault or twinning at the bimetallic interfaces (Fig. 2). TEM-EDX mapping images of Au@Pd–SiO_2_ (Fig. 3a and b) and Pd@Au–SiO_2_ (Fig. 3d and e) showed that Pd and Au atoms are homogeneously distributed in both the materials. As seen from the TEM-EDX mapping image in Fig. 3b for the material Au@Pd–SiO_2_, the intensity of green dots representing Au atoms was higher at the centre while the intensity of white dots representing Pd atoms is more at the surface.

**Fig. 4:**
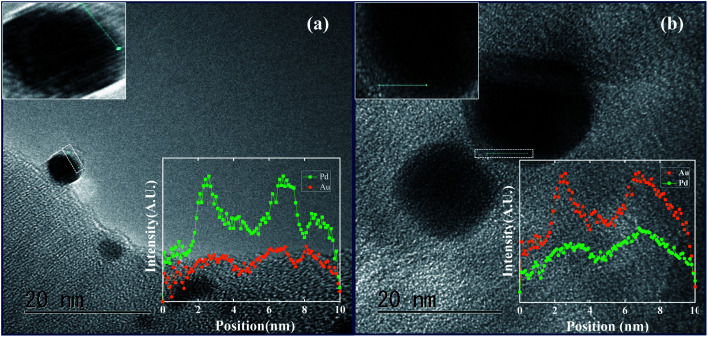
TEM micrograph and EDS line scanning profile of a single particle (highlighted on inset) of (a) Au@Pd–SiO_2_ and (b) Pd@Au–SiO_2_.

A magnified cross-sectional view of an individual particle in the inset (Fig. 3b) presented a clear idea of the differences in intensities of the metal atoms and highlights large patches of Au atoms at the core accompanied by small patches of Pd atoms at the shell. The fact was further corroborated by a TEM-EDX line scanning profile diagram obtained along the edges (Fig. 4a) as well as across the core (Fig. S7a†) which shows a galvanic type structure with an Au rich centre and a Pd rich boundary (Fig. 4a). On the other hand, for the material Pd@Au–SiO_2_, the TEM-EDX mapping images (Fig. 3e) showed that the white dots representing Pd atoms are more intense at the centre than the green dots for Au atoms. The magnified cross-sectional image of a single particle (inset, 3e) demonstrated a dense and pristine spherical core composed of Pd atoms surrounded by a thin layer of Au atoms. As anticipated, the TEM-EDX line profile diagrams (Fig. 4b and S7b†) also confirmed a Pd rich centre engulfed by an Au rich boundary, a typical anti-galvanic type structure. Similar to TEM-EDX mapping, FESEM-EDX images (Fig. S6†) of both Pd@Au–SiO_2_ and Au@Pd–SiO_2_ showed the presence of Au and Pd atoms on the silica support. Thus, by controlling the addition sequence of metal salts both galvanic (Au_core_–Pd_shell_) and anti-galvanic type (Pd_core_–Au_shell_) Au–Pd NPs could be accessed.

**Fig. 5:**
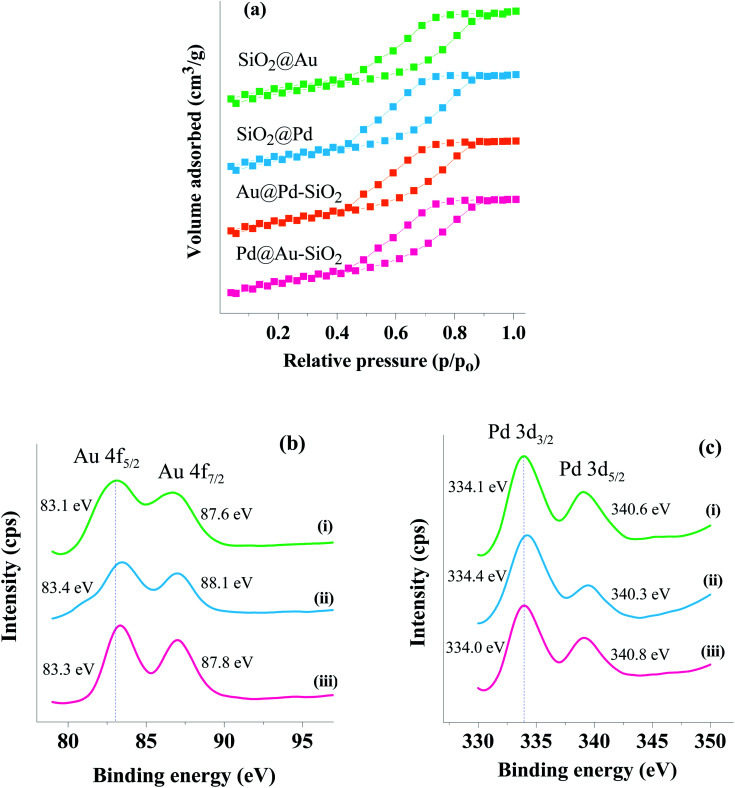
(a) N_2_-sorption isotherms of the nanoparticle incorporated silica-based materials: SiO_2_@Au, SiO_2_@Pd, Au@Pd–SiO_2_ and Pd@Au–SiO_2_, (b) XPS spectra of (i) SiO_2_@Au, (ii) Pd@Au–SiO_2_ and (iii) Au@Pd–SiO_2_ showing the Au 4f_5/2_ and 4f_7/2_ states and (c) XPS spectra of (i) SiO_2_@Pd, (ii) Au@Pd–SiO_2_ and (iii) Pd@Au–SiO_2_ 3d_5/2_ and 3d_3/2_ showing Pd 3d_5/2_ and 3d_3/2_ states.

N_2_ adsorption–desorption isotherms were measured for the materials at 77 K and the physical parameters were compared with those for the parent support. The N_2_-sorption measurements for the materials with respect to the neat support showed an isotherm with the type IV hysteresis loop characteristic of mesoporous materials. A prominent decrease in the surface area compared to the neat support was observed for Au@Pd–SiO_2_ (Fig. 5a) whereas only a marginal decrease was recorded for the SiO_2_@Au, SiO_2_@Pd and Pd@Au–SiO_2_ materials (Fig. 5a).

Nevertheless, the materials exhibited a gradual decrease in surface area as well as pore volume on moving from the neat support through monometallic to bimetallic nanoparticles (Table 1). This steady decrease can be attributed to the generation of mono/bimetallic nanoparticles within the pores of the silica matrices.

**Table 1:** A comparative study of the surface area, pore volume and metal content of the core–shell nanoparticles with their neat support

Sample	*S*_BET_ a [m^2^ g^−1^]	Pore volume^b^ [cm^3^ g^−1^]	Metal content^c^ [% wt/wt]
2-Diphenylphosphinoethyl functionalized silica gel	500.9	0.476	—
SiO_2_@Au	411.3	0.882	0.96
SiO_2_@Pd	423.3	0.663	0.84
Au@Pd–SiO_2_	380.0	0.633	0.78(Pd), 0.91(Au)
Pd@Au–SiO_2_	408.6	0.445	0.82(Pd), 0.89(Au)

a BET surface area was measured from the N_2_-sorption isotherm at 77 K.

b Total pore volume was measured at *P*/*P*_0_ = 0.98.

c Metal content of the materials as measured from ICP-AES analysis.

The Au@Pd–SiO_2_ and Pd@Au–SiO_2_ nanoparticles were further subjected to XPS analysis (Fig. 5b and c) to confirm the metallic nature of Au and Pd. The XPS peak values are also compared to those of the monometallic counterparts *i.e.*, SiO_2_@Au [Fig. 5b(i)] and SiO_2_@Pd [Fig. 5c(i)]. For Au@Pd–SiO_2_, the XPS peaks for Pd are observed at 334.4 and 340.3 eV [Fig. 5c(ii)] corresponding to Pd 3d_3/2_ and Pd 3d_5/2_ electronic states coherent with the value for Pd(0) species.^35^ For Au, peaks were observed at 83.3 and 87.8 eV [Fig. 5b(iii)] corresponding to Au 4f_5/2_ and 4f_7/2_ respectively coherent with the value for Au(0) species.^45^ Similarly, for Pd@Au–SiO_2_, XPS peaks were observed at 334 and 340.8 eV for Pd 3d_3/2_ and Pd 3d_5/2_ [Fig. 5c(iii)], and at 83.4 and 88.1 eV for Au 4f_5/2_ and Au 4f_7/2_ [Fig. 5b(ii)] respectively. Notably compared to monometallic Au NPs, in Au@Pd–SiO_2_, where Au was present as the core and Pd as the shell, peaks due to Au 4f_5/2_ and 4f_7/2_ were marginally shifted to higher energies (*ca.* +0.2 eV), while in Pd@Au–SiO_2_, where Au was present as the shell, relatively higher shifts were noticed (+0.3 and +0.5 eV for 4f_5/2_ and 4f_7/2_ respectively), consistent with a change in the electronic environment presumably due to easy charge transfer between the well-organized Au–Pd interfaces induced by the phosphine donors in the ligand. A similar type of effect resulting in increment of binding energies due to the presence of phosphine donor ligands has been reported in the literature.^35,46^ Similar trends were also noticed for Pd, where shifts of +0.3 and −0.3 eV were evident for 3d_3/2_ and 3d_5/2_ electronic states respectively. It may be worth noting that XPS analysis can also be used to determine the thickness of shells in core–shell materials up to a depth of 10 nm.^47^ Accordingly, we have calculated the shell thickness of Au@Pd–SiO_2_ and Pd@Au–SiO_2_ and values of 2.54 and 1.46 nm were obtained respectively. However, compared to the shell thickness determined by TEM analysis (Fig. 1a and b) the values for the respective materials were 4.6 and 2.8% lower. The relatively high mismatch obtained in the case of Au@Pd–SiO_2_ compared to Pd@Au–SiO_2_ may be attributed to the inhomogeneous shell thickness and dissymmetric core–shell interfaces. Such an inhomogeneous shell with a core displaced from the particle center can often lead to an underestimation of the shell thickness.

The amount of Au and Pd present on the synthesized materials was estimated by ICP-AES analysis and was found to be 0.91 and 0.78 for Au@Pd–SiO_2_ and 0.89 and 0.82 wt% for Pd@Au–SiO_2_ respectively (Table 1).

In one of our previous reports, we have laid special emphasis on the role of ligands with different donors *viz.* N-, P- and S-atoms for the synthesis of Pd nanomaterials with different sizes, shapes and dimensions. Among the various ligands, the phosphine donor ligand demonstrated Pd NPs with exceptional stability, purity and dispersity. The Pd NPs exhibited sizes averaged at 6 nm and a *d*-spacing value of 0.223 nm with reticular planes extending throughout the structure without any stacking or fault. Additionally, XPS studies revealed that the interaction of P-donor atoms on the ligand-functionalized silica matrices induces a small positive charge on the Pd metal surface. A cumulative influence of these factors can help utilize these Pd NPs as suitable cores for the deposition of a second metal. In this respect, a thick layer of Au was homogeneously deposited onto the preformed Pd cores leading to Pd_core_–Au_shell_ nanoparticles.

## Catalytic activity

### Hydrogenation of nitroarenes

Aromatic chloroamines are widely used as important intermediates in the chemical industry for the production of pharmaceuticals, agrochemicals, polymers and dyes.^48^ Currently, access to those amines occurs mainly through reduction of appropriate nitro compounds using molecular H_2_ as the reductant. Although molecular H_2_ is an environmentally benign reducing agent, in some cases, particularly under high pressure, the use of gaseous H_2_ could make the system potentially explosive and thus limit its industrial scale utility.^49^ Moreover, gaseous hydrogen at higher pressures can also lead to hydrodechlorination reaction through C–Cl bond scission to produce undesired anilines in high yields thereby affecting the selectivity.^35^ Another important route to access chloroamines is transfer hydrogenation of nitroarenes which utilizes hydrogen supplied from a co-solvent, usually an alcohol such as 2-propanol. However, the production of hydrogen from 2-propanol usually requires higher temperatures and longer reaction times as observed in recent reports.^50^ On the contrary, addition of hydrogen from sources other than molecular hydrogen and alcohols (*e.g.* ammonia borane,^51^ formic acid,^52^ hydrazine hydrate,^53,54^*etc.*) has emerged as much safer alternatives in recent times. Among those hydrogen sources, hydrazine hydrate is particularly promising as it produces N_2_ as the sole by-product thereby ensuring higher safety.

**Scheme 2:**
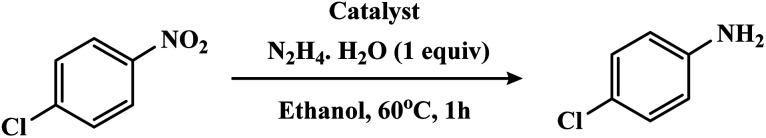
Hydrogenation of 4-chloronitrobenzene (4-CNB) to 4-chloroaniline (4-CAN) with different catalysts under liquid phase conditions.

Literature evidence suggests that bimetallic systems involving the use of a second metal with Pd or Au often improve their catalytic efficacies for various organic transformations.^55,56^ However, such improvements are usually at the crucial cost of selectivity. Today, a number of catalytic systems, including metal nanoparticles of Pd or Au (*e.g.* PdNP@CeO_2_,^57^ AuNP@TiO_2_,^58^*etc.*) are known to promote this hydrogenation. However, such systems usually suffer from unsatisfactory chemoselectivity, particularly in cases of functionalized nitro substrates that contain groups like carbonyls, halogens, *etc.* owing to the side reactions of over-hydrogenation/dehalogenation.^53,59^ Thus, the search for more promising heterogeneous catalysts that can produce targeted amines through safer hydrogen sources without compromising with the chemoselectivity is highly desirable. Hence, we have tested the hydrogenation prowess of our bimetallic Au–Pd materials, taking 4-chloronitrobenzene (4-CNB) as the model substrate (2 mmol) and ethanol (5 ml) as the solvent with hydrazine hydrate (1 equivalent) at 60 °C for 1 h (Scheme 2).

**Fig. 6:**
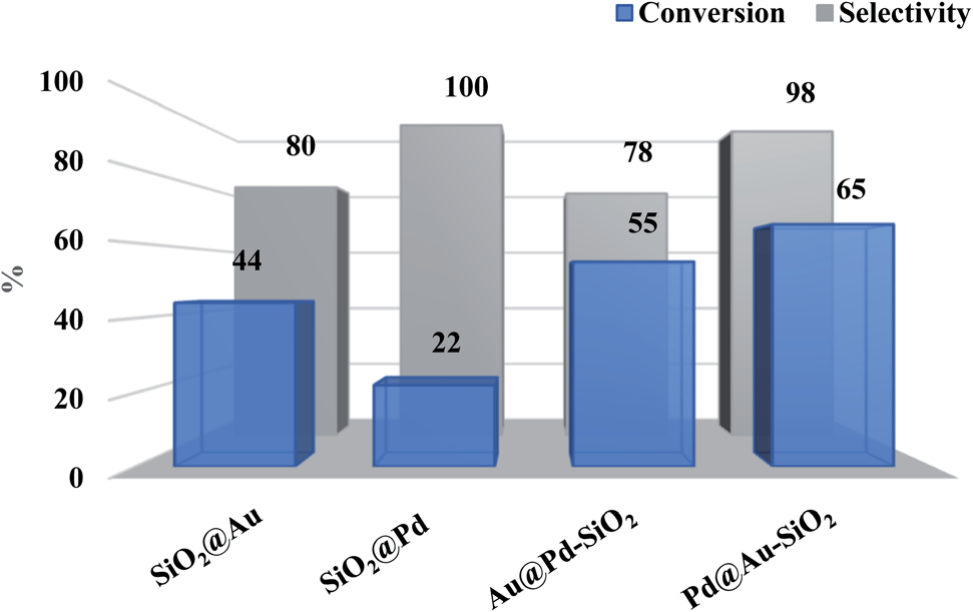
Comparison of conversions and selectivity obtained for different types of catalysts: SiO_2_@Au (Au: 0.28 mol%); SiO_2_@Pd (Pd: 0.28 mol%); Au@Pd–SiO_2_ (Au: 0.16 mol%, Pd: 0.12 mol%); Pd@Au–SiO_2_ (Au: 0.13 mol%, Pd: 0.15 mol%) (metal concentrations in the catalysts were determined in terms of ICP-AES analysis).

To illustrate the synergistic effects, we have also tested the catalytic efficacies of the corresponding silica-supported monometallic Au and Pd NPs. As shown in Fig. 6, the monometallic Pd catalyst, Pd@SiO_2_, gave only 22% of 4-chloroaniline (4-CAN) with a selectivity of 100%, while the monometallic Au catalyst, Au@SiO_2_, showed slightly higher activity (conversion: 44%) with a moderate drop in selectivity (80%). Interestingly, use of bimetallic catalysts (Pd@Au–SiO_2_ or Au@Pd–SiO_2_) significantly improved the activity, demonstrating the synergistic influence between the two metals. Under the same set of experimental conditions, the Pd@Au–SiO_2_ catalyst having Pd_core_–Au_shell_ structure displayed superior activity and selectivity over the Au@Pd–SiO_2_ catalyst having the Au_core_–Pd_shell_ structure. This higher activity of the Pd@Au–SiO_2_ catalyst could be attributed to the uniform and well-defined Au–Pd bimetallic interfaces compared to the non-uniform interfaces in Au@Pd–SiO_2_.^34^ Homogeneous interfaces may lead to pronounced ligand, ensemble and strain effects^60^ that govern the mechanism of synergism in a bimetallic material. Higher synergy will promote greater activity while lower synergy produces a diminishing effect on activity.

**Fig. 7:**
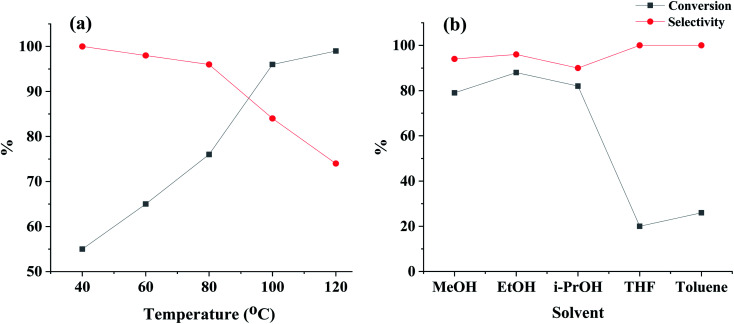
(a) Temperature optimization for the Pd@Au–SiO_2_ catalyst [Au (0.13 mol%), Pd (0.15 mol%)] with 4-CNB as the model substrate (2 mmol) and ethanol (5 ml) as the solvent for 1 h and (b) solvent optimization studies under the above conditions at 80 °C.

Considering the superior catalytic performance of Pd@Au–SiO_2_ over others, we have applied it for further optimization studies. Indeed, a temperature optimization study with Pd@Au–SiO_2_ (Fig. 7a) revealed that both the activity and selectivity are temperature dependent. It has been seen that an increase in temperature resulted in an increase in conversion, although a little drop in selectivity was noticed. The optimum temperature was found to be 80 °C when 76% conversion was achieved with a 4-CAN selectivity of 96%.

Interestingly, by doubling the hydrazine hydrate concentration (from 1 mmol to 2 mmol) the conversion could be increased to 88% while maintaining a selectivity of 96%. Indeed, apart from temperature and hydrazine–hydrate concentration, the choice of solvent also had some vital impact on our catalytic system (Fig. 7b). Among the solvents screened, ethanol was found to be the most effective, while THF was the least effective. To determine the best catalytic conditions, additional screenings such as catalyst : substrate ratio, effect of hydrogen source, *etc.* were performed with our model system (Table S1†). Important conclusions can be drawn from these studies. Firstly, on increasing the catalyst : substrate ratio, a decrease in both conversion (72%) and selectivity (90%) was observed (Table S1,† entry 8). Secondly, an increase in the reaction time led to almost stoichiometric conversion (98%) while at the same time brought down the selectivity (84%) owing to extended exposure to longer time intervals and subsequent circumcision of the C–Cl bond from the preformed 4-CAN (Table S1,† entry 7). Finally, the reaction was also screened in the presence of different hydrogen sources under similar experimental conditions to explore the rates of reduction exhibited by these agents (Table S1,† entries 1–4, 6, 9–12).

After systemic optimization of a number of reaction variables, it was observed that a reaction time of 1 h coupled with a temperature of 80 °C, 0.28 mol% of Pd@Au–SiO_2_ [Au (0.13 mol%), Pd (0.15 mol%)], 2 mmol of model substrate (4-CNB) and 2 equivalents of hydrazine hydrate in ethanol (5 ml) demonstrated best results and were chosen for substrate scope evaluation.

To explore the scope and limitations of the present methodology, different nitroarenes were examined under the optimized reaction conditions and the results are presented in Table 2. Nitroarenes containing electron-donating, electron-neutral as well as electron-withdrawing substituents mostly at the *para*-position could provide desired anilines in excellent yields and selectivity. Indeed, this high conversion and selectivity were also maintained with halogen-substituted nitroarenes without any noticeable dehydrohalogenated products (2a–e). Thus, our result is quite satisfactory as we could achieve the desired amines with selectivity >95% without compromising with the activity.

**Table 2:** Substrate scope of Au–Pd catalyzed hydrogenation reactions^a^^,^^b^^,^^c^

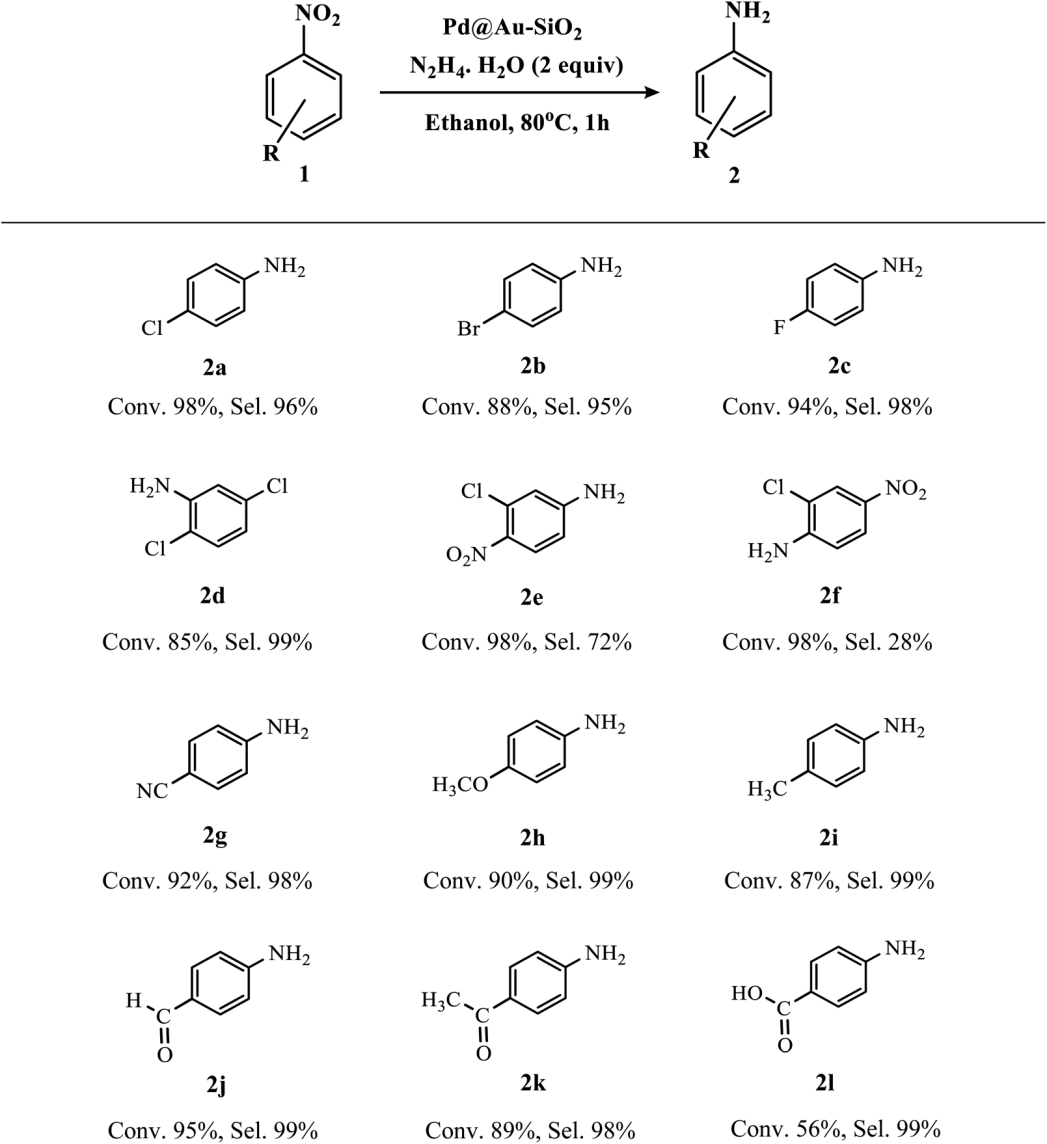

a Reaction conditions: substrate (2 mmol), ethanol (5 ml), Pd@Au–SiO_2_ [Au (0.13 mol%), Pd (0.15 mol%)], hydrazine–hydrate (2 equivalents) at 80 °C for 1 h.

b Determined by GC-MS analysis and aniline (AN) was formed as the sole by-product.

c MS-spectra available in the ESI.

In fact, most of the reported catalytic systems showed either relatively low conversion,^61^ or selectivity <90%.^62,63^ Remarkably, the subtle nature of the catalyst was unveiled with a slightly challenging substrate like 2,5-dichloronitrobenzene, as both the –Cl groups were retained in the product forming 2,5-dichloroaminobenzene as the sole product (2d). Interestingly, substituted dinitrobenzene compounds like 1-chloro-2,5-dinitrobenzene underwent hydrogenation to furnish a monoaminated product instead of the diamine. The reaction was regioselective for 3-chloro-4-nitroaniline (2e) instead of 2-chloro-4-nitroaniline (2f) which might be attributable to the intramolecular hydrogen bonding observed in the case of the 2-chloro derivative. Functionalized nitroarenes containing reducible moieties are considered to be difficult substrates to hydrogenate chemoselectively.^64,65^ However, we are gratified to see that our bimetallic Au–Pd catalyst can effectively perform hydrogenation of compounds like 4-nitrobenzaldehyde and 4-nitroacetophenone and produce 4-aminobenzaldehyde (commonly known as PABA, used as a biodegradable pesticide and as a UV-filter in sunscreens)^66^ (2j), and 4-aminoacetophenone (2k) almost quantitatively with selectivity >98%, without affecting the easily reducible carbonyl groups. Similarly, 4-nitrobenzoic acid was converted to 4-aminobenzoic acid (2l) with moderate conversion and excellent selectivity.

In order to ascertain the superiority of our catalyst, a comparative study was performed with reported Au- or Pd NP based catalysts immobilized onto different supports (Table 3). Clearly, Pd-based catalysts are of prominent interest in this regime. Most of these catalysts, just like in our case do not involve the use of bases for activation. A number of these catalysts involve high temperatures (*e.g.*, Pd/AlPO_4_/SiO_2_, Au@Pt/TiO_2_, Au–Pd/Mo_2_C), prolonged reaction times (*e.g.*, Co@Pd/NC, Pd/ZrP), high metal loadings (*e.g.*, Au–Pd/Mo_2_C), drastic conditions (*e.g.*, Au–Sn/SiO_2_) and deleterious solvents (*e.g.*, Au–Sn/SiO_2_, Au–Ni/TiO_2_). Although, the activity and selectivity of catalysts such as G-NiPd, PdNi/mCN and PdCu@MWCNT seem promising, lengthy and multistep synthetic procedures hinder their industrial applicability. Use of moisture sensitive precursors further adds to the complicacy in their handling procedures. Hence, it is apparent from Table 3 that Pd@Au–SiO_2_ is much more superior to the reported catalysts.

**Table 3:** A comparative catalytic study of the hydration of nitroarenes using various bimetallic catalysts^a^

Catalyst	Conditions	Efficiency	Selectivity	Ref.
Pd/AlPO_4_/SiO_2_	Et_3_N, 90–95 °C, neat, 2–5 h	Low yields for arenes bearing EDG	Good selectivity on: ketone, ester	67
			Poor selectivity on: halide	
Pd–Au/TiO_2_	No base, N_2_H_4_·H_2_O, R.T., neat, 10–60 min	Low yields for arenes bearing 3-iodo, amino and biaryl groups	—	55
Pd@Au–SiO_2_	No base, N_2_H_4_·H_2_O, 80 °C, ethanol, 1 h	Good yields for arenes bearing EWG and EDG (56–98%)	Good selectivity on halide, nitrile, aldehyde, ketone, acid	This work
Pd/ZrP	No base, 40–60 °C, ethanol, 2–24 h	Good yields for EDG (99–99%)	Good selectivity on: ester, nitrile, chlorine and double bond	68
PdNi/mCN	No base, R.T., ethanol : H_2_O (3 : 7), 40–60 min	Good yields (84–100%)	Good selectivity on: halide (Cl, Br, I)	69
PdCu@MWCNT	No base, NaBH_4_, R.T., methanol : H_2_O (3 : 7), 10–60 min	Good yields for arenes bearing EDG (12–99%)	Good selectivity on: heteroaryl, nitrile, chlorine, bromine and alkyl group	70
Co@Pd/NC	No base, H_2_ (1 bar), R.T., EtOAc, 1–22 h, (1.5% Au, 0.01% Pt)	Good yields for arenes bearing EDG (93–99%)	Good selectivity on: ester, nitrile, chlorine and double bond	71
			Long intervals on: bromine	
Au@Pt/TiO_2_	No base, H_2_ (8 bar), 90–95 °C, toluene, 1–22 h, (1.5% Au, 0.01% Pt)	Good yields for 3-nitrostyrene (94%)	Not tested for other substrates	72
G-NiPd	No base, NH_3_BH_3_, R.T., methanol : H_2_O (3 : 7), 5–15 min	Good yields for arenes bearing EDG (>99%)	Good selectivity on: heteroaryl, fluorene, bromine and aliphatic groups	73
			Long intervals on: 3-amino group	
Au–Sn/SiO_2_	No base, H_2_ (13 bar), 80–100 °C, toluene, 1.5 h, (0.55% Au, 1% Sn)	Good yields for arenes bearing nitrobenzene (29.1–99%) and 3-nitrostyrene (24.2–99%)	Good selectivity on: amide, amino, aldehyde, nitrile and chlorine bond	74
Au–Pd/Mo_2_C	No base, H_2_ (1 bar), 220 °C, gas-phase, 1 h, (0.84% Au, 11.7% Pd)	Good yields for nitro, chloro and nitrile (100%)	Good selectivity on: halide and nitrile	75

a EDG = electron donating group, EWG = electron withdrawing group, Ref. = references.

### Reusability and heterogeneity

As reusability is one of the most important features of any supported nanomaterial, we have performed a recyclability test of our catalyst, Pd@Au–SiO_2_, taking *p*-CNB as the substrate under optimized conditions. After performing the reaction, the catalyst was separated by centrifugation, washed with ^*i*^PrOH–H_2_O (1 : 1), dried and then used for subsequent runs. Our results showed that the catalyst could be used at least 5 times without noticeable changes in activity and selectivity. The slight drop in activity might be due to unavoidable handling loss of the sample during recycling experiments.

**Fig. 8:**
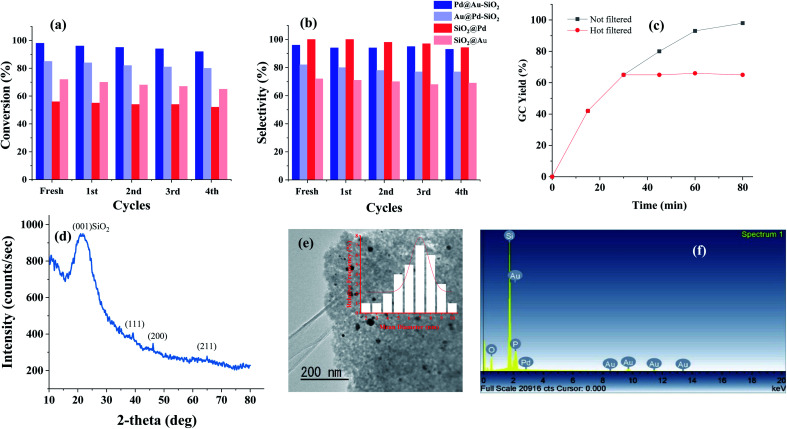
Recyclability tests for the catalysts performed up to the 5th run corresponding to both (a) conversion and (b) selectivity. (c) Hot filtration test for 4-CNB hydrogenation using Pd@Au–SiO_2_. (d) Powder-XRD of the material. (e) TEM image of Pd@Au–SiO_2_ after the 5th run with the corresponding size histogram (inset) and (f) TEM-EDX spectra of the catalyst recovered after recyclability tests performed up to the 5th run.

For complete evaluation of reusability, systematic recycling experiments for all the catalysts under the optimized set of conditions were performed which demonstrated very little change in activity or selectivity (Fig. 8a and b). The powder X-ray diffraction pattern of the material obtained after the 5th run showed all three prominent reflections corresponding to the (111), (200) and (211) planes of fcc Au and Pd (Fig. 8d). In fact, TEM (Fig. 8e) and TEM-EDX studies (Fig. 8f) of the recovered catalyst (Pd@Au–SiO_2_) after the 5th run showed that there is virtually no change in the surface morphology suggesting that the catalyst could be reused for more runs. To check the heterogeneity of our catalyst, the hot filtration test was performed using 4-CNB as a substrate. The reaction was performed with Pd@Au–SiO_2_ as the catalyst under a hydrazine–hydrate concentration of 2 equivalents and a temperature of 80 °C in ethanol. The reaction time was shortened from 1 h to a period of 15 minutes. After completion of the reaction, the catalyst was carefully separated from the reaction mixture and the filtrate was subsequently analyzed using a GC-MS which showed a conversion of 49% with a selectivity of 100%. Interestingly, when the filtrate was stirred for one more hour under similar conditions, no increase in conversion was observed (Fig. 8c) suggesting that there was no leaching of Au or Pd into the solution. In fact, the ICP-AES analysis for the catalyst after the fifth cycle exhibited almost negligible loss of Au (0.011 wt%) and Pd (0.019 wt%) into the solution thus demonstrating the heterogeneous nature of the core–shell material.

### Nitrile hydration

**Scheme 3:**
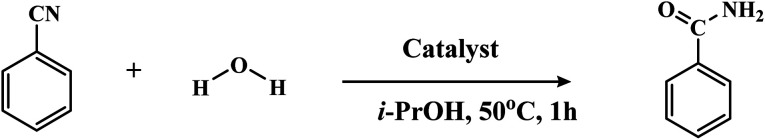
Hydration of benzonitrile to benzamide with different catalysts under liquid phase conditions.

**Fig. 9:**
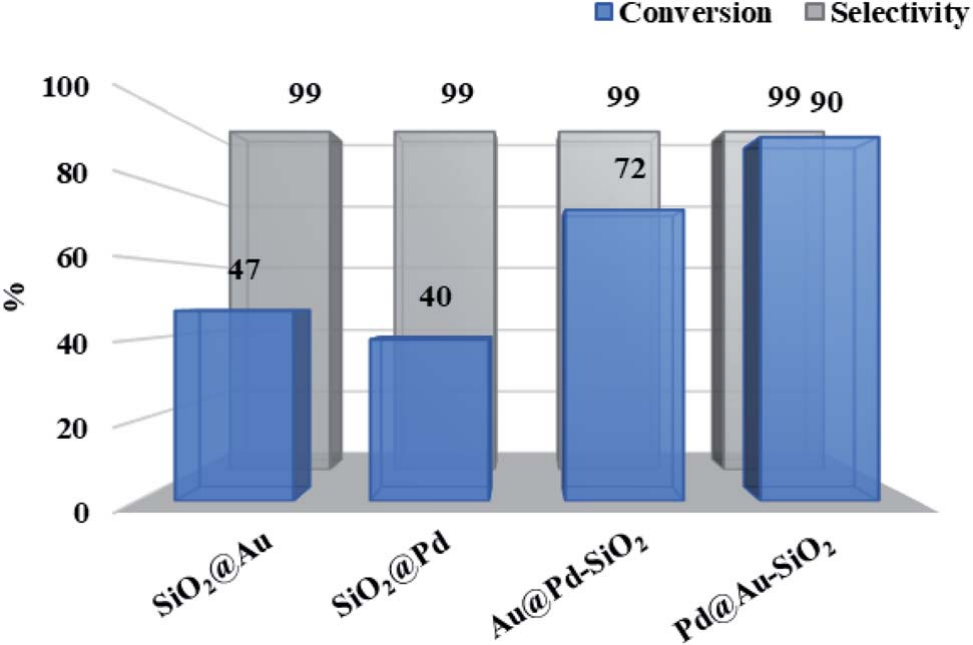
Comparison of conversion and selectivity obtained for different catalysts: SiO_2_@Au (Au: 0.2 mol%); SiO_2_@Pd (Pd: 0.2 mol%); Au@Pd–SiO_2_ (Au: 0.12 mol%, Pd: 0.08 mol%); Pd@Au–SiO_2_ (Au: 0.09 mol%, Pd: 0.11 mol%) (metal concentrations in the catalysts were determined by ICP-AES analysis).

Transition metal mediated hydration of nitriles to amides is one of the most atom-economical methods for the conversion of aromatic nitriles to corresponding amides.^76,77^ A number of noble-metal based catalysts including nanoparticles of Au and Pd are known for this transformation.^78^ However, to the best of our knowledge, there is not a single report on the use of bimetallic Au–Pd catalysts for the hydration of nitriles. Thus, with the catalyst at hand, we have tested the activity of our bimetallic core–shell materials for the nitrile hydration reaction. The hydration of benzonitrile was used as a test reaction for initial screening studies and the reactions were conducted taking aqueous ^*i*^PrOH (1 : 1 mixture) as solvent at 50 °C for 1 h (Scheme 3). Like hydrogenation, in this case also we have compared the catalytic efficacies of the monometallic Au and Pd NPs. As seen in Fig. 9, the monometallic Au or Pd NPs show more or less comparable results, however the catalytic activity significantly improved when we used bimetallic catalysts Pd@Au–SiO_2_ or Au@Pd–SiO_2_, coherent with the synergistic influence between Au and Pd. Like hydrogenation, the Pd_core_–Au_shell_ catalyst (Pd@Au–SiO_2_) displayed superior activity over the Au_core_–Pd_shell_ catalyst (Au@Pd–SiO_2_).

It needs to be mentioned that because of the poor solubility of nitriles in water, mixed aqueous solvents are often preferred in the nitrile hydration reaction. To find the best solvent system for our reaction, a range of mixed aqueous media were screened (Table S2†). Almost comparable results were obtained with MeOH–H_2_O and EtOH–H_2_O at 50 °C (entries 2 and 5). However, anticipating better solubility of nitriles in ^*i*^PrOH over MeOH, we have chosen the ^*i*^PrOH–H_2_O mixture for further optimization studies (entry 7).

As seen from Table S2,† (entries 7–10), the catalytic activity is also dependent on the ratio of ^*i*^PrOH and H_2_O. The best condition was obtained when ^*i*^PrOH and H_2_O were used in a 1 : 1 ratio. On decreasing the ^*i*^PrOH–H_2_O ratio the reactivity declined, presumably due to a decrease in the solubility of substrates with decreasing ^*i*^PrOH count in the reaction mixture. An increase in the reaction time resulted in a pragmatic increase in conversion (Table S3,† entries 3–5). Furthermore, on increasing the catalyst : substrate ratio, a subsequent increase in conversion was observed (Table S3,† entry 6). In addition to routine screening, a temperature optimization study was performed to explore the effects of temperature on conversion and selectivity (Table S3†). It was observed that a marginal increase in temperature to 60 °C leads to almost quantitative conversion (95%) without any variation in selectivity (Table S3,† entry 5). Any further increase in the temperature had no adverse effect on either activity or selectivity (Table S3,† entries 7 and 8). In fact, no trace of acid by-product was recorded in any of the performed optimization experiments.

With the optimized reaction conditions (^*i*^PrOH : H_2_O (1 : 1), 60 °C, 1 h, 0.5 mmol of substrate and 0.2 mol% of Pd@Au–SiO_2_ [Au (0.09 mol%), Pd (0.11 mol%)]), we further examined the scope of our catalyst for the hydration of a range of nitriles to amides and the results are presented in Table 4. It was evident that most of the ortho-substrates demonstrated low conversions compared to the *meta*- and *para*-analogues. A similar trend was observed in a representative study by Cadierno *et al.* irrespective of the nature of the aromatic ring (aryl, pyridyl, piperidyl, thienyl, *etc.*).^79^ Electron-negative functionalities (4d–f and 4j) exhibited good-to-excellent conversions (80–99%) with excellent selectivity (99%). Conversely, nitriles containing electron donating groups like –CH_3_ (4g) and –OCH_3_ (4i) exhibited moderate conversions with excellent selectivity >99%. Likewise, the catalyst was also screened for nitrogen-containing heteroaromatic substrates (4a–c). Depending on the relative position of the –CN group and the heteroatom, noticeable differences in the activities were observed. Almost quantitative conversions were achieved for 2-cyanopyridine and 4-cyanopyridine furnishing picolinamide (4b) and isonicotinamide (4c), respectively, after about 30 min. However, a much longer reaction time (1 h) was required to obtain nicotinamide (4a) from 3-cyanopyridine in high yield. Plausible reasons for this difference in reactivity incline towards resonance effects since in (4a) the nitrile carbon has a reduced electrophilicity (negative charges are always located on the carbon atoms of the pyridine ring *vs.* N atoms in the case of 4b and 4c), and therefore the nucleophilic addition of water at this position is less favoured.^79^ Nicotinamide (4a), a form of vitamin B_3_, is used as a common dietary supplement^80^ while picolinamide (4c), used as a template for the synthesis of molecular imprinting polymers,^81^ is particularly important for pharmaceutical and industrial applications. Smooth conversion as well as selectivity were recorded in the case of haloaromatic substrates (–F, –Cl and –Br) (4d–f). Credibly, our catalyst could also tolerate benzylic nitrile derivatives (4l–m) yielding the desired product with moderate-to-good conversion and excellent selectivity.

**Table 4:** Substrate scope of Au–Pd catalyzed nitrile hydration using alkyl and aryl cyanides^a^^,^^b^^,^^c^

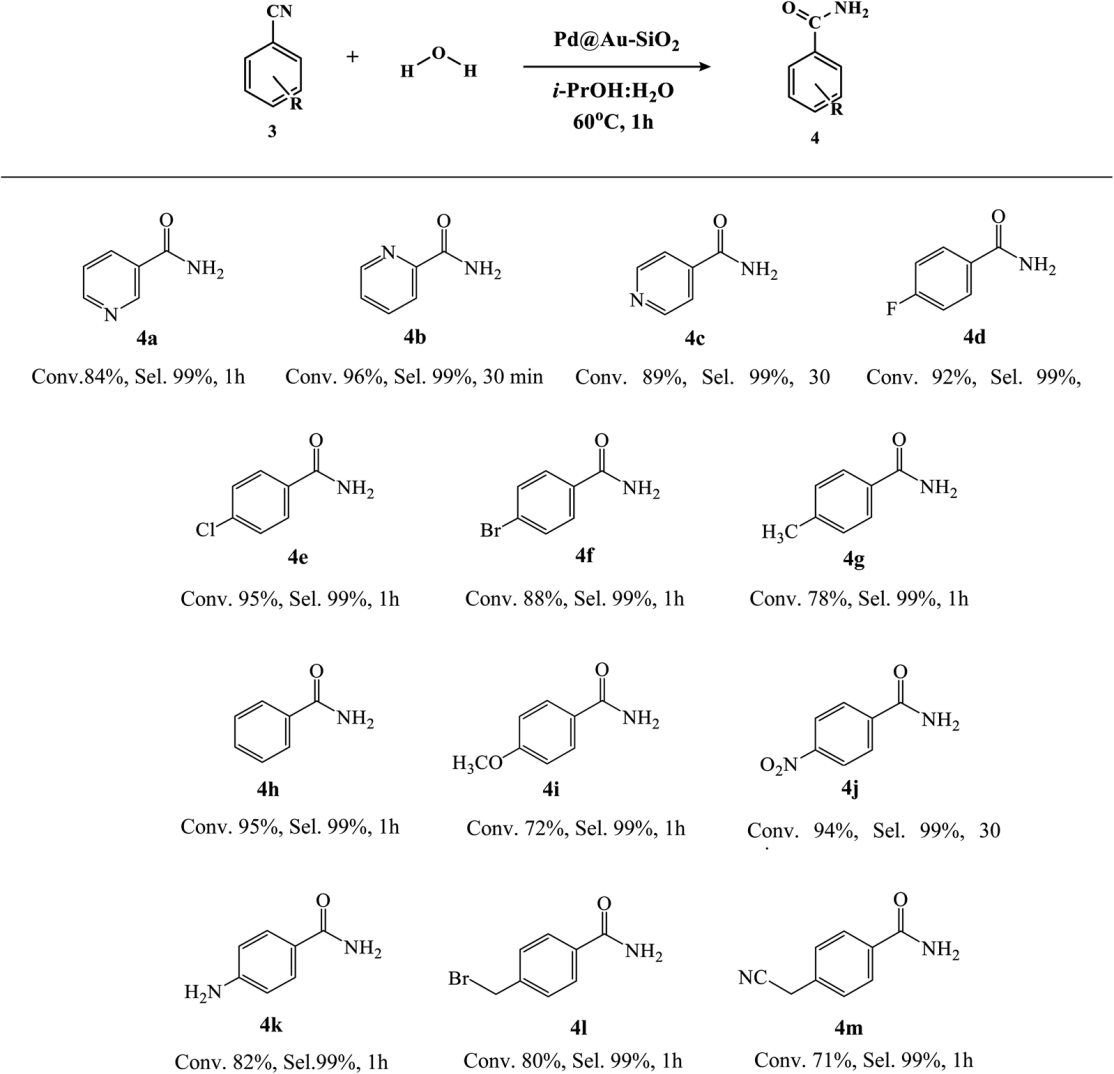

a Reaction conditions: substrate (0.5 mmol), H_2_O (0.089 mmol), Pd@Au–SiO_2_ [Au (0.09 mol%), Pd (0.11 mol%)], ^*i*^PrOH–H_2_O (1 : 1, 4 ml) at 60 °C for 1 h.

b Determined by GC-MS analysis.

c MS-spectra available in the ESI.

### Kinetic studies

**Fig. 10:**
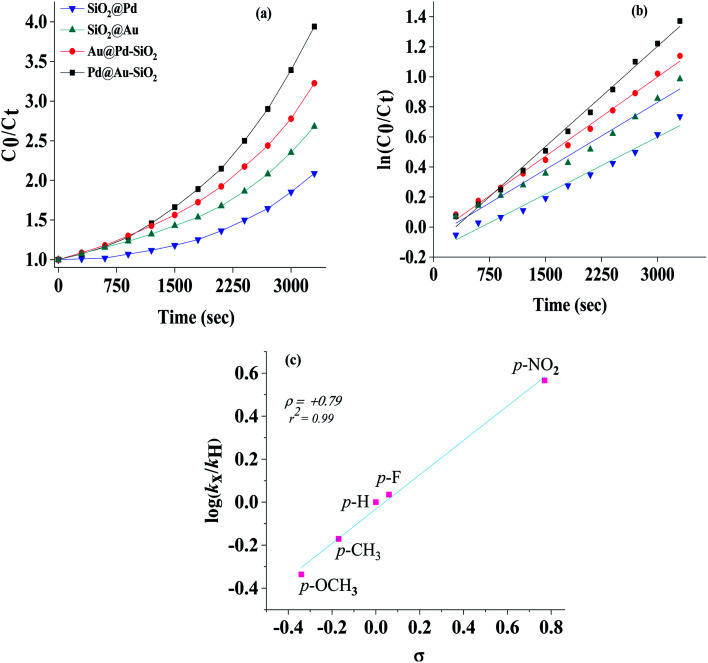
(a) *C*_0_/*C*_*t*_*vs.* time and (b) ln(*C*_0_/*C*_*t*_) *vs.* time plots for the hydration of nitriles with Pd@Au–SiO_2_ under similar experimental conditions, where *C*_0_ and *C*_*t*_ are the concentrations of benzonitrile at time 0 and *t* respectively (c) Hammett plot for the hydration of benzonitrile and *p*-substituted benzonitriles, conditions: nitrile (0.5 mmol), Pd@Au–SiO_2_ (12.5 mg), H_2_O/*i*-PrOH (1 : 1).

The kinetics for the conversion of benzonitrile to benzamide was investigated by monitoring the reaction at different time intervals and plots of conversion *versus* time are presented in Fig. 10 which in most cases followed a parabolic curve. A linear fit of first order was observed in each case as was evidenced from the stacked plots of *C*_0_/*C*_*t*_*versus* time (Fig. 10a) and ln(*C*_0_/*C*_*t*_) *versus* time (Fig. 10b), where *C*_0_ and *C*_*t*_ are the concentrations of benzonitrile at time 0 and *t* respectively. From the slope of the plot, the first order rate constants were calculated and found to correspond to the following order of efficacies, Pd@Au–SiO_2_ (4.448 × 10^−4^ s^−1^) > Au@Pd–SiO_2_ (3.617 × 10^−4^ s^−1^) > SiO_2_@Au (2.979 × 10^−4^ s^−1^) > SiO_2_@Pd (2.536 × 10^−4^ s^−1^). The relationship between the relative rates and the Hammett parameter (*s*) for the hydration of various nitriles by Pd@Au–SiO_2_ was examined.

The order of reactivity for hydration of benzonitrile was 4-NO_2_ (*k*_X_/*k*_H_ = 2.71) > 4-F (1.29) > 4-H (1.00) > 4-CH_3_ (0.82) > 4-OCH_3_ (0.82). The relative rates [log(*k*_X_/*k*_H_)] were plotted against the substituent constant *σ* (Fig. 10c). There was a fairly good linearity between log(*k*_X_/*k*_H_) and *σ* (*r*^2^ = 0.99), and the *σ* slope of the linear line in Fig. 10c gives a Hammett, *ρ* (rho) value of +0.79. The typical positive *ρ* value indicated that the reaction is largely favoured by the presence of electron withdrawing groups at the *para*-position.

### Reusability and heterogeneity

**Fig. 11:**
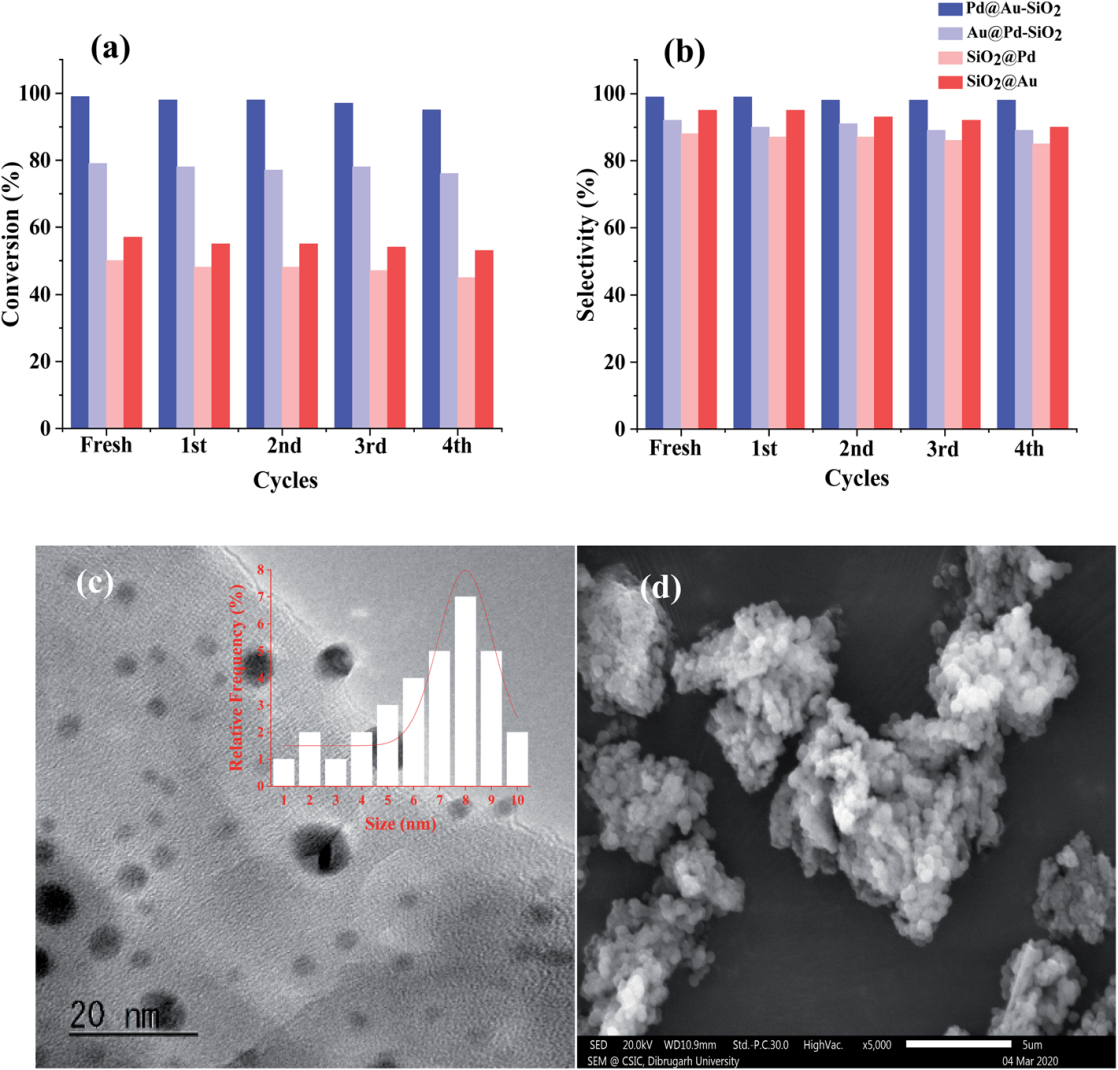
Reusability tests for the catalysts performed up to the 5th run corresponding to both (a) conversion and (b) selectivity. (c) HRTEM image and size histogram (inset, 100 particles per site count) of Pd@Au–SiO_2_ after the 5th run with the corresponding (d) FESEM micrograph of the material.

In order to test the heterogeneity of the material, Pd@Au–SiO_2_ reusability tests were performed using benzonitrile as the model substrate. To evaluate this property the catalyst was carefully separated from the reaction mixture by centrifugation, filtration and subsequent washing with ^*i*^PrOH–H_2_O (1 : 1), and it was dried and reused for successive runs (Fig. 11). The tests were performed maintaining the previously used stoichiometric ratio (Table S3,† entry 5). This result suggested that the catalyst could be used for five successive runs without compromising much with the activity. A marginal loss in selectivity was observed in subsequent runs which could be assigned to the gradual blockage of some of the active sites in the silica matrix as well as due to unavoidable weight losses encountered during recycling.

Reusability tests were also performed for the other three catalysts under the optimized reaction conditions which exhibited minimal change in activity or selectivity. The extent of Au/Pd leaching into the solution has been estimated using ICP-AES analysis of the filtrate after the 5th run. The analysis demonstrated a negligible loss of 0.012 wt% in Pd and 0.02 wt% in Au content.

To check the heterogeneity of the catalyst, hot filtration test was performed and benzonitrile was used as the model substrate. The reaction was stopped after 15 minutes and the conversion was recorded to be 24% (analyzed by GC-MS). The reaction mixture was separated by filtration under hot conditions and the filtrate was allowed to stir continuously for another hour. The conversion was found to be almost constant which confirmed the heterogeneous nature of the catalyst. The morphology of the catalyst after the fifth run was examined using HRTEM analysis (Fig. 11c) which exhibited a fine size distribution (inset) just like the fresh material. The HRTEM micrograph showed that the surface morphology of the catalyst remained almost intact without any particle agglomeration. This result is in agreement with the notion that the material is catalytically stable to be used for yet more catalytic runs.

To apprehend the advantages of our catalyst, a comparative study was carried out with literature reported Au- or Pd NP based catalysts immobilized onto different supports (Table 5). Although, the activity and selectivity of some of those catalysts are very similar, in most cases the metal content was radically high ranging from 2–10 mol%. Moreover, most of the reported systems are monometallic in nature and either required long intervals [*e.g.*, Pd(OAc)_2_]^83^ or high temperatures (*e.g.*, Pd-PVP, Pd/C-500ox),^84,88^ thus confronting with the sustainability issue. Hence, it is apparent from Table 5 that our bimetallic Pd@Au–SiO_2_ catalyst is much more superior to the reported catalysts.

**Table 5:** A comparative catalytic study of the hydration of benzonitrile to benzamide under liquid phase conditions

Catalyst	Conditions^a^	Metal content (mol%)	Conversion (%)	Selectivity (%)	References
Au@Pd–SiO_2_	50 °C, 1 h	Au (0.09), Pd (0.11)	90^b^	99^b^	This work^a^
Pd@Au–SiO_2_	50 °C, 1 h	Au (0.12), Pd (0.08)	72^b^	99^b^	This work^a^
Pd(NO_3_)_2_	50 °C, 10 min	2–10	94	—	82
Pd(OAc)_2_	70 °C, 24 h	5	88	—	83
Pd(OAc)_2_/Sc(OTf)_3_	30 °C, 12 h	Sc (3), Pd (1.5)	80	—	84
Pd_3_P_0.95_ QDs	90 °C, 4 h	1	86	—	85
Pd-PVP	180 °C, 12 h, CuSO_4_	5	99	99	86
Au–TiO_2_-VS	60 °C, 5 h	2	>99	99	87
Pd/C-500ox	95–140 °C, 24 h	2	99	96	88

a Reaction conditions: benzonitrile (0.5 mmol), H_2_O (0.089 mmol), ^*i*^PrOH–H_2_O (1 : 1, 4 ml).

b Determined by GC-MS analysis.

## Conclusion

In summary, we have methodically synthesized Au–Pd core–shell nanoparticles supported on 2-diphenylphosphinoethyl functionalized silica gel by a sequential reduction technique. Merely, by controlling the addition sequence of metal salts, both galvanic (Au_core_–Pd_shell_) and anti-galvanic (Pd_core_–Au_shell_) type core–shell nanoparticles were synthesized. We have mainly focused our study on two intriguing aspects, firstly, the differences in structural morphology of core–shell nanoparticles introduced through fabrication *via* the preemptive ligand assisted method and secondly, the catalytic performances of the materials have been measured in terms of their respective synergistic effects for two significant reactions *viz.* hydrogenation of nitroarenes to amines and hydration of nitriles to amides under mild conditions. Remarkably, with one of our catalysts, we could achieve high conversion as well as excellent selectivity for both the aforementioned reactions. The protocol reported herein for the synthesis of Au–Pd core–shell nanoparticles anchored to functionalized silica for efficient hydrogenation to amines and nitrile hydration to amides may open new avenues in synthetic methodologies, and environmental and catalytic research in future.

## Conflicts of interest

There are no conflicts to declare.

## Supplementary Material

NA-003-D1NA00489A-s001
