# Supplementary material for: Bimetallic Au–Pd nanoparticles supported on silica with a tunable core@shell structure: enhanced catalytic activity of Pd(core)–Au(shell) over Au(core)–Pd(shell)
Source: Nanoscale Adv. 2021 Aug 9;3(18):5399–416. doi: 10.1039/d1na00489a (PMC9417894; doi:10.1039/d1na00489a)
Supplement: NA-003-D1NA00489A-s001 [file NA-003-D1NA00489A-s001.pdf]

Electronic Supplementary Material (ESI) for Nanoscale Advances.
This journal is © The Royal Society of Chemistry 2021

# Bimetallic Au-Pd nanoparticles supported on silica with tunable core@shell structure: Enhanced catalytic activity of Pd(core)-Au(shell) over Au(core)-Pd(shell)

*Gauravjyoti D. Kalita*[a], *Podma P. Sarmah*[a], *Golap Kalita*[b], *Pankaj Das*[a]*

[a]Department of Chemistry, Dibrugarh University, Dibrugarh, Assam, India, 786004.
[b]Department of Physical Science and Engineering, Nagoya Institute of Technology (NiTech), Nagoya, Aichi, Japan-466-8555.

## Supporting Information

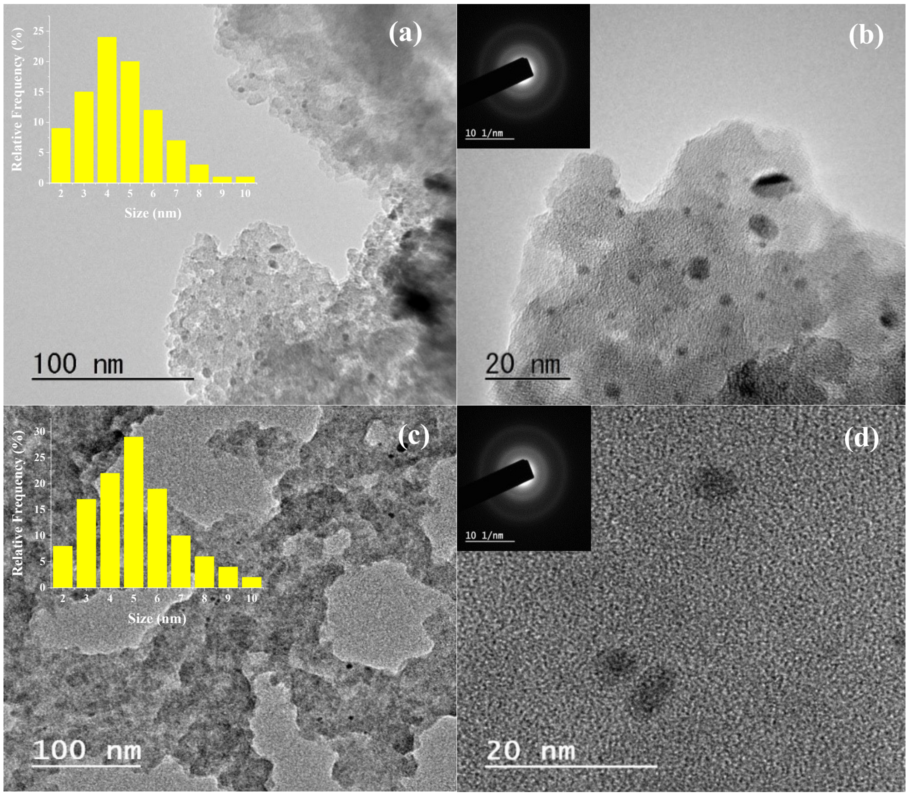


**Fig. S1 (a)** TEM image and size-distribution histogram (50 particles per site count) of $SiO_2$@Au with corresponding **(b)** HRTEM image and SAED pattern depicted on inset; **(c)** TEM image and size-distribution histogram (50 particles per site count) of $SiO_2$@Pd with corresponding **(d)** HRTEM image and SAED pattern depicted on inset

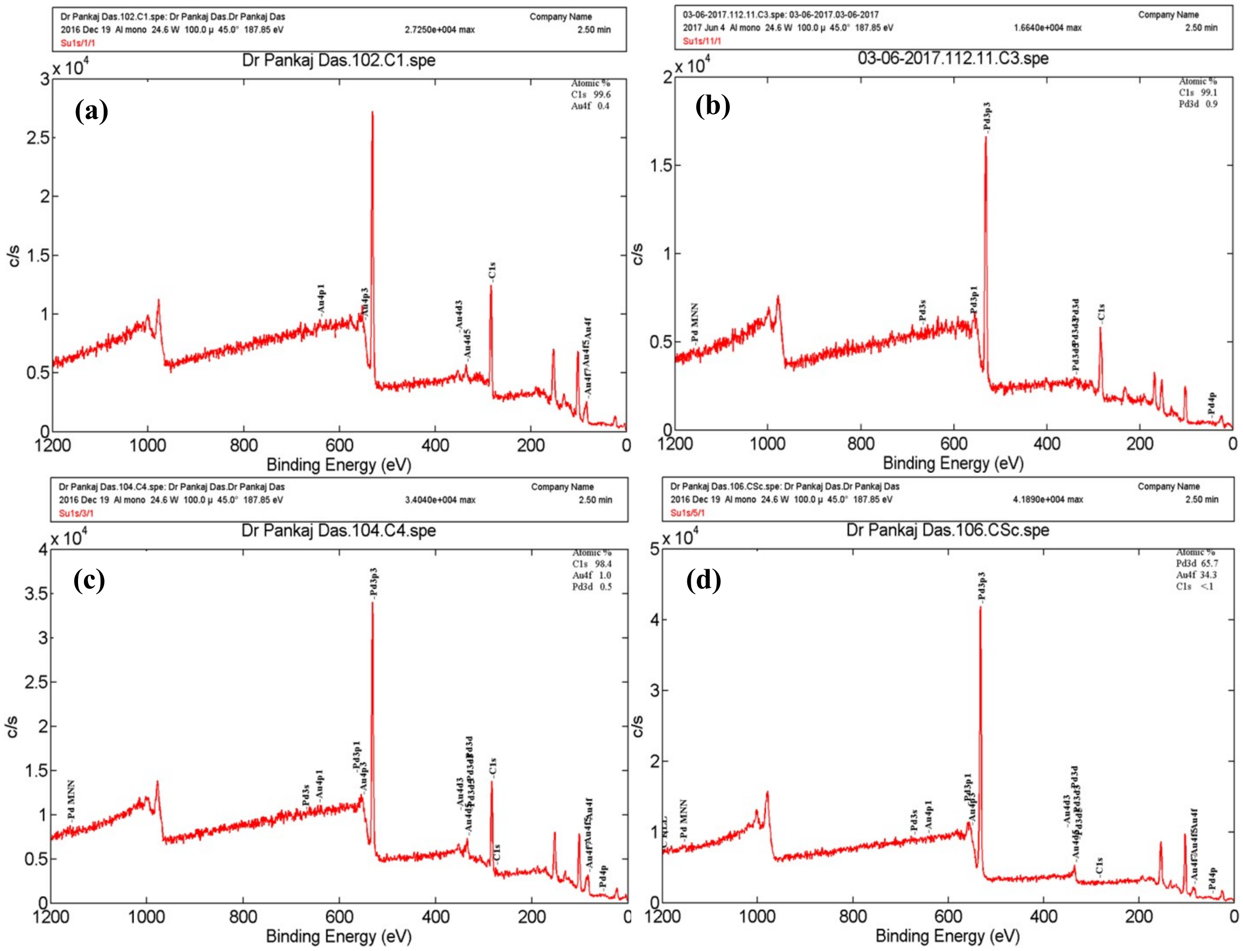


**Fig. S2** XPS survey spectrum of the as prepared **(a)** $SiO_2$@Au, **(b)** $SiO_2$@Pd **(c)** Pd@Au-$SiO_2$ and **(d)** Au@Pd-$SiO_2$ materials

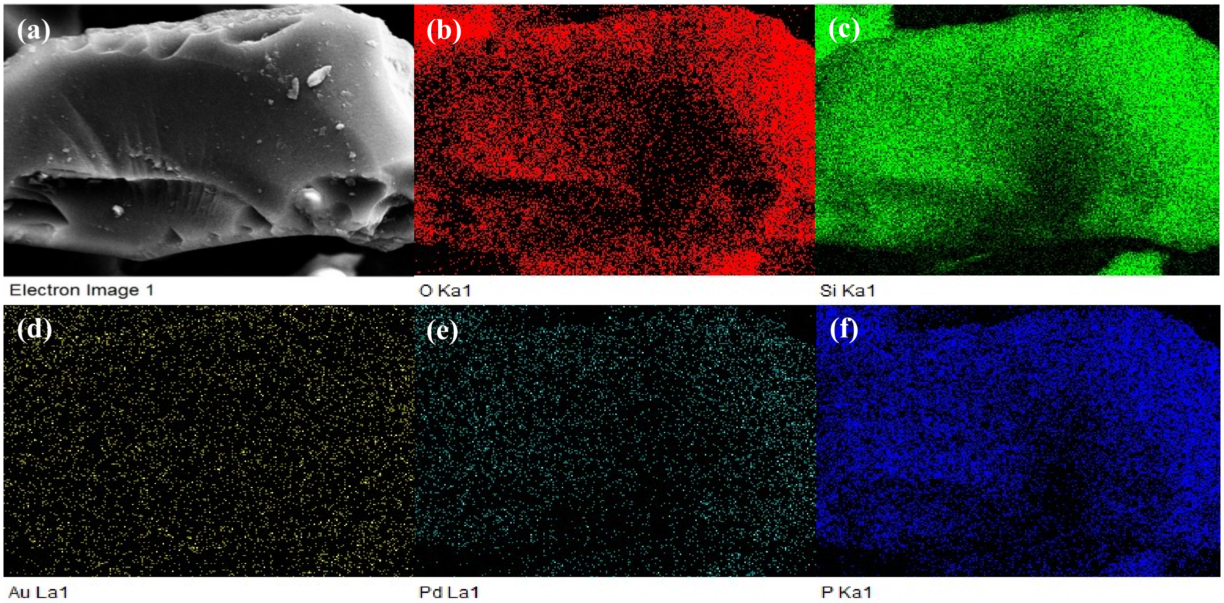


**Fig. S3** FESEM-EDS images **(a)** electron image with corresponding elemental distributions **(b)** O Ka 1 **(c)** Si Ka1 **(d)** Au La1 **(e)** Pd La1 **(f)** P Ka1 of nanoparticle incorporated Pd@Au-$SiO_2$ material

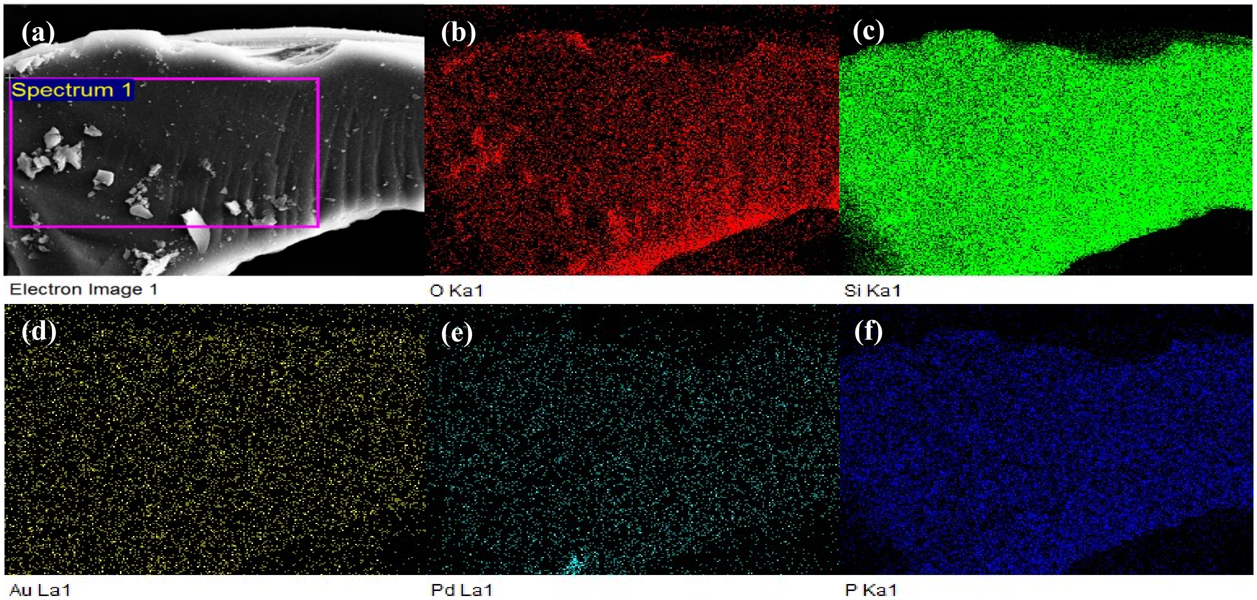


**Fig. S4** FESEM-EDS images **(a)** electron image with corresponding elemental distributions **(b)** O Ka 1 **(c)** Si Ka1 **(d)** Au La1 **(e)** Pd La1 **(f)** P Ka1 of nanoparticle incorporated Au@Pd-$SiO_2$ material

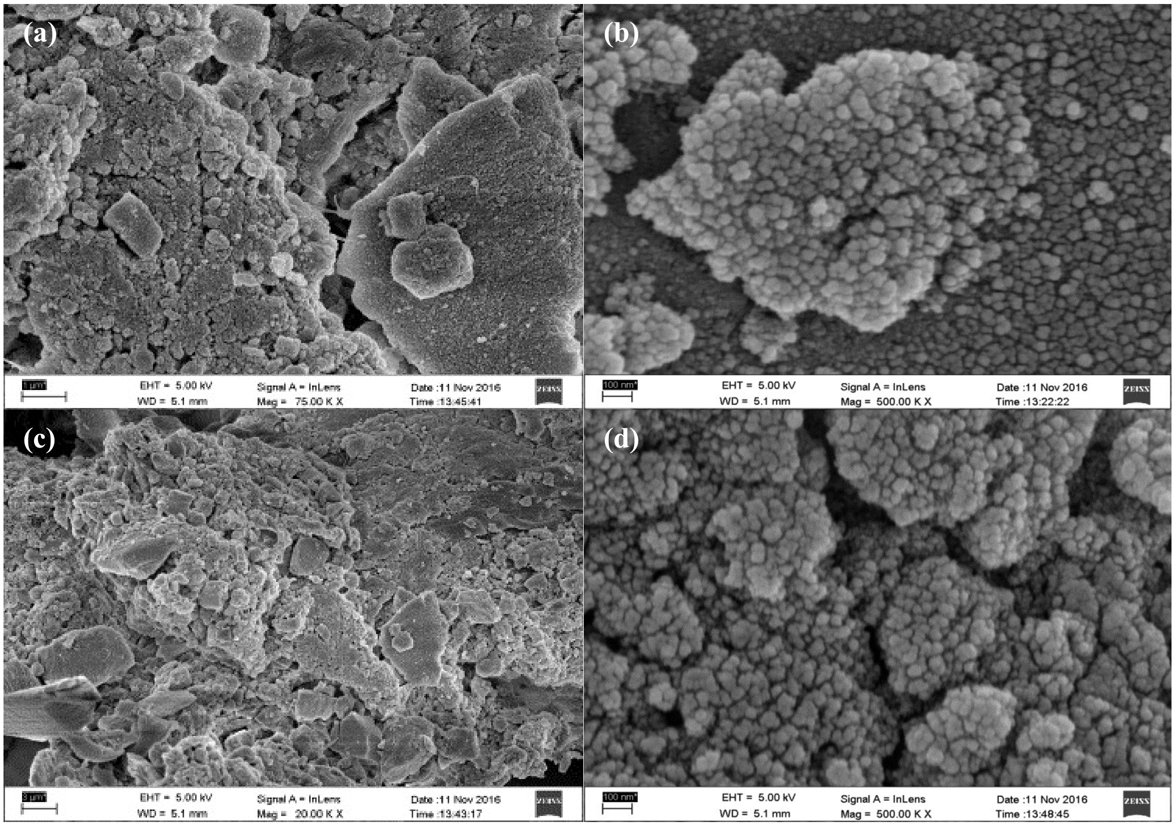


**Fig. S5** FESEM images **(a)** and **(b)** for Pd@Au-$SiO_2$; and **(c)** and **(d)** for Au@Pd-$SiO_2$

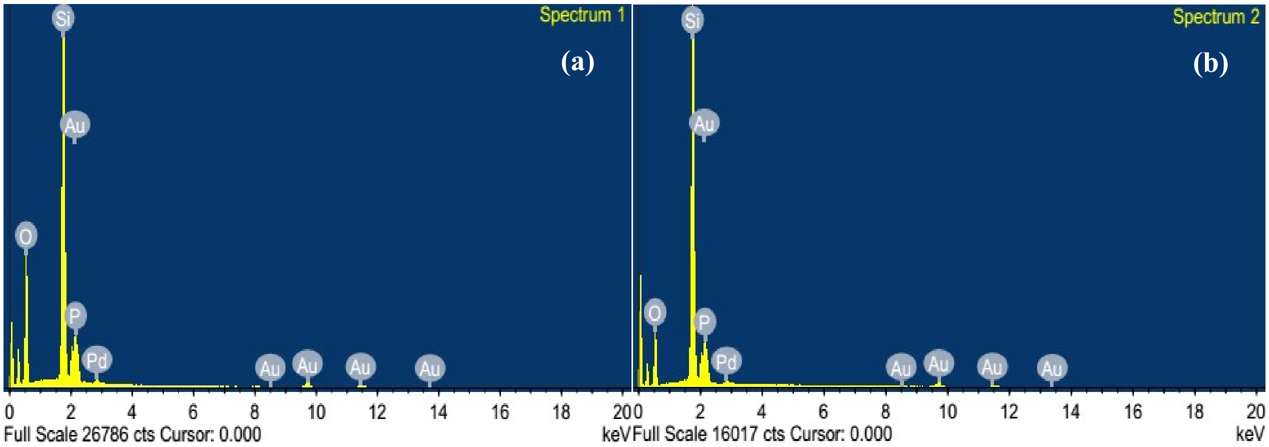


**Fig. S6** FESEM-EDX spectra of **(a)** Pd@Au-$SiO_2$ and **(b)** Au@Pd-$SiO_2$

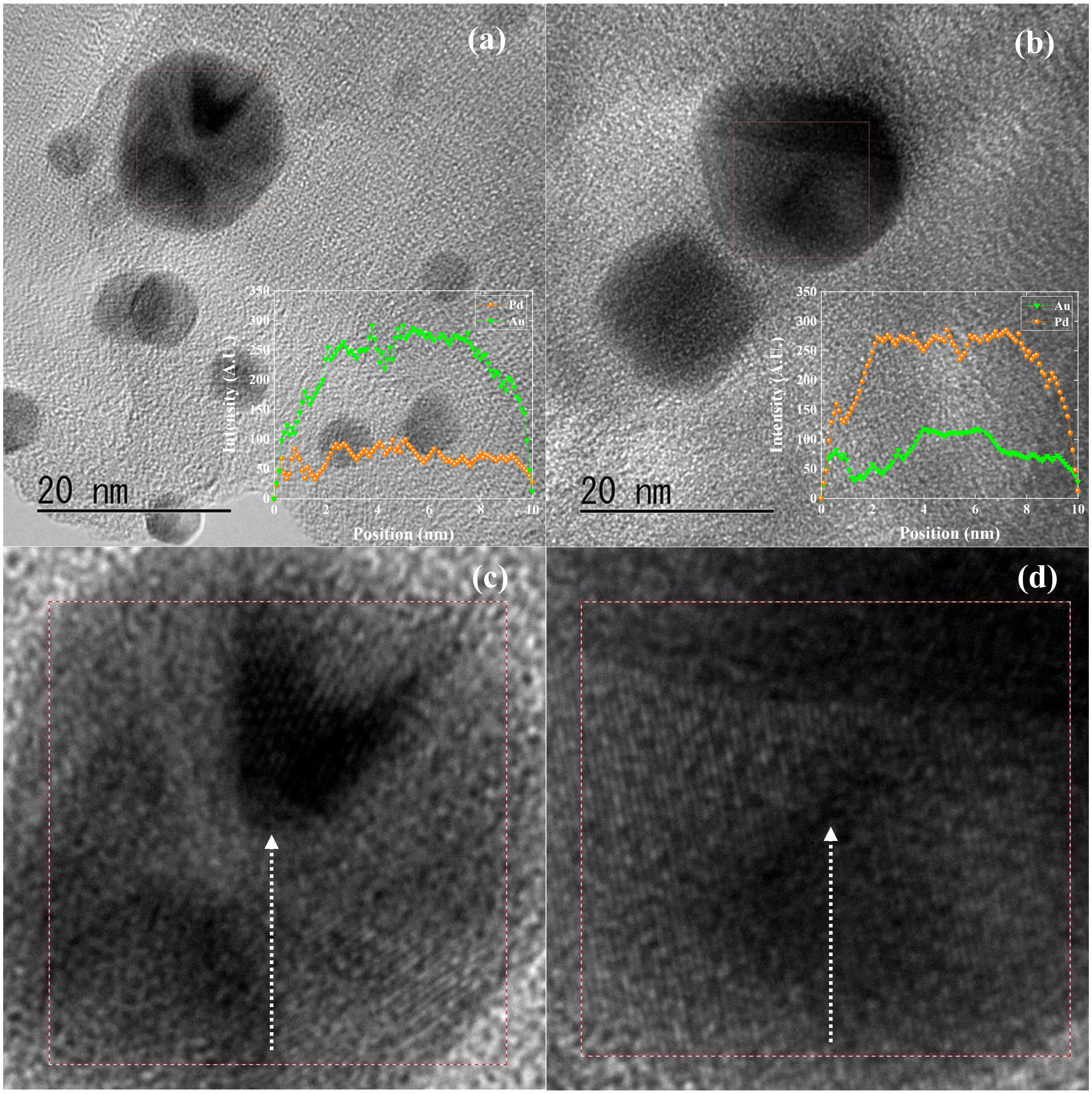


**Fig. S7** TEM micrographs and line scan profiles (inset) for **(a)** Au@Pd-$SiO_2$ and **(b)** Pd@Au-$SiO_2$ along with the respective scanned cross-sections for **(c)** Au@Pd-$SiO_2$ and **(d)** Pd@Au-$SiO_2$

**Table S1** Hydrogenation of 4-CNB with Pd@Au-$SiO_2$ core-shell nanoparticles as catalysts under liquid phase conditions [a]

| Entry | Solvent | Reductant | Substrate (mmol) | Time (h) | Conversion [%] [b] | Selectivity [%] [b, c] |
|---|---|---|---|---|---|---|
| 1 | EtOH | $NH_3$. $BH_3$ | 2 | 5 | 44 | 86 |
| 2 | EtOH | $HCOONH_4$ | 2 | 1 | 18 | 99 |
| 3 | EtOH | HCOOH | 2 | 1 | 20 | 99 |
| 4 | EtOH | $H_2$ | 2 | 1 | 4 | 99 |
| 5 | EtOH | $N_2H_4$. $H_2O$ | 2 | 0.5 | 42 | 99 |
| 6 | EtOH | $N_2H_4$. $H_2O$ | 2 | 1 | 88 | 98 |
| 7 | EtOH | $N_2H_4$. $H_2O$ | 2 | 2 | 98 | 88 |
| 8 | EtOH | $N_2H_4$. $H_2O$ | 4 | 1 | 76 | 96 |
| 9 | EtOH | Ethanol | 2 | 5 | NR | - |
| 10 | *i*-PrOH | *i*-PrOH | 2 | 5 | NR | - |
| 11 | EtOH | $NaBH_4$ | 2 | 3 | 35 | 80 |
| 12 | EtOH | Ascorbic acid | 2 | 24 | 2 | 99 |

**[a]** Reaction conditions: Pd@Au-$SiO_2$ [Au (0.13 mol%), Pd (0.15 mol%)], ethanol (5 mL), 400 rpm at 80°C, **[b]** Determined by GC-MS analysis, **[c]** Aniline (AN) was formed as the sole by-product **[d]** $H_2$ gas pressure was maintained at 1 bar.

**Table S2** Comparison of catalytic efficiency of Pd@Au-$SiO_2$ with different solvents for hydration of benzonitrile [a]

| Entry | Solvent (ratio) | Time (h) | Conversion (%) [c] | Selectivity (%) [c] |
|---|---|---|---|---|
| 1 | $H_2O$ (Neat) | 24 | - | 99 |
| 2 | EtOH: $H_2O$ (1:1) | 10 | 64 | 99 |
| 3 | EtOH: $H_2O$ (1:10) | 10 | Trace | 99 |
| 4 | DMSO | 8 | 62 | 99 |
| 5 | MeOH: $H_2O$ (1:1) | 5 | 89 | 99 |
| 6 | MeOH: $H_2O$ (1:10) | 12 | 60 | 99 |
| 7 | $^i$PrOH: $H_2O$ (1:1) | 1 | 90 | 99 |
| 8[b] | $^i$PrOH: $H_2O$ (1:1) | 1 | 95 | 99 |
| 9 | $^i$PrOH: $H_2O$ (1:5) | 1 | 36 | 99 |
| 10 | $^i$PrOH: $H_2O$ (1:10) | 1 | Trace | 99 |

**[a]** Reaction conditions: Benzonitrile (0.5 mmol), Pd@Au-$SiO_2$ [Au (0.09 mol%), Pd (0.11 mol%)], solvent (4 mL), 400 rpm, 50°C, **[b]** 60°C, **[c]** Determined by GC-MS analysis.

**Table S3** Hydration of benzonitrile with Pd@Au-$SiO_2$ core-shell nanoparticles as catalysts under liquid phase conditions [a]

| Entry | Substrate (mmol) | Temperature (°C) | Time (min) | Conversion [%] [b] | Selectivity [%] [b] |
|---|---|---|---|---|---|
| 1 | 0.5 | 40 | 60 | 78 | 99 |
| 2 | 0.5 | 50 | 60 | 90 | 99 |
| 3 | 0.5 | 60 | 20 | 34 | 99 |
| 4 | 0.5 | 60 | 40 | 69 | 99 |
| 5 | 0.5 | 60 | 60 | 95 | 99 |
| 6 | 1 | 60 | 60 | 85 | 99 |
| 7 | 0.5 | 70 | 60 | 97 | 99 |
| 8 | 0.5 | 80 | 60 | 96 | 99 |

**[a]** Reaction conditions: Pd@Au-$SiO_2$ [Au (0.09 mol%), Pd (0.11 mol%)], $^i$PrOH: $H_2O$ (1:1) (4 mL) and 400 rpm, **[b]** Determined by GC-MS analysis

**Calculation of shell widths from XPS peak intensities**

High-resolution XPS spectra were taken (Figure 5b and 5c), from which the peak areas of C 1s, Pd 3d and Au 4f were measured and the ratios $A_C/A_{Pd}$, $A_C/A_{Au}$ and $A_{Pd}/A_{Au}$ were calculated.

where, $A_C$ = Absorption intensity of $C_{1s}$ peak,

$A_{Pd}$ = Absorption intensity of $Pd_{3d_{3/2}}$ peak, and

$A_{Au}$ = Absorption intensity of $Au_{4f_{5/2}}$ peak.

This reference method was adopted from a recent work by Yan et al.[1] that involved a similar calculation[2] with XPS binding energies for thiol functionalized Au NPs.

## GC-Mass spectra of Products

### Hydrogenation

### 4-chloroaniline

Instrument : GCMSD
Sample Name: HM27
Misc Info :
Vial Number: 1

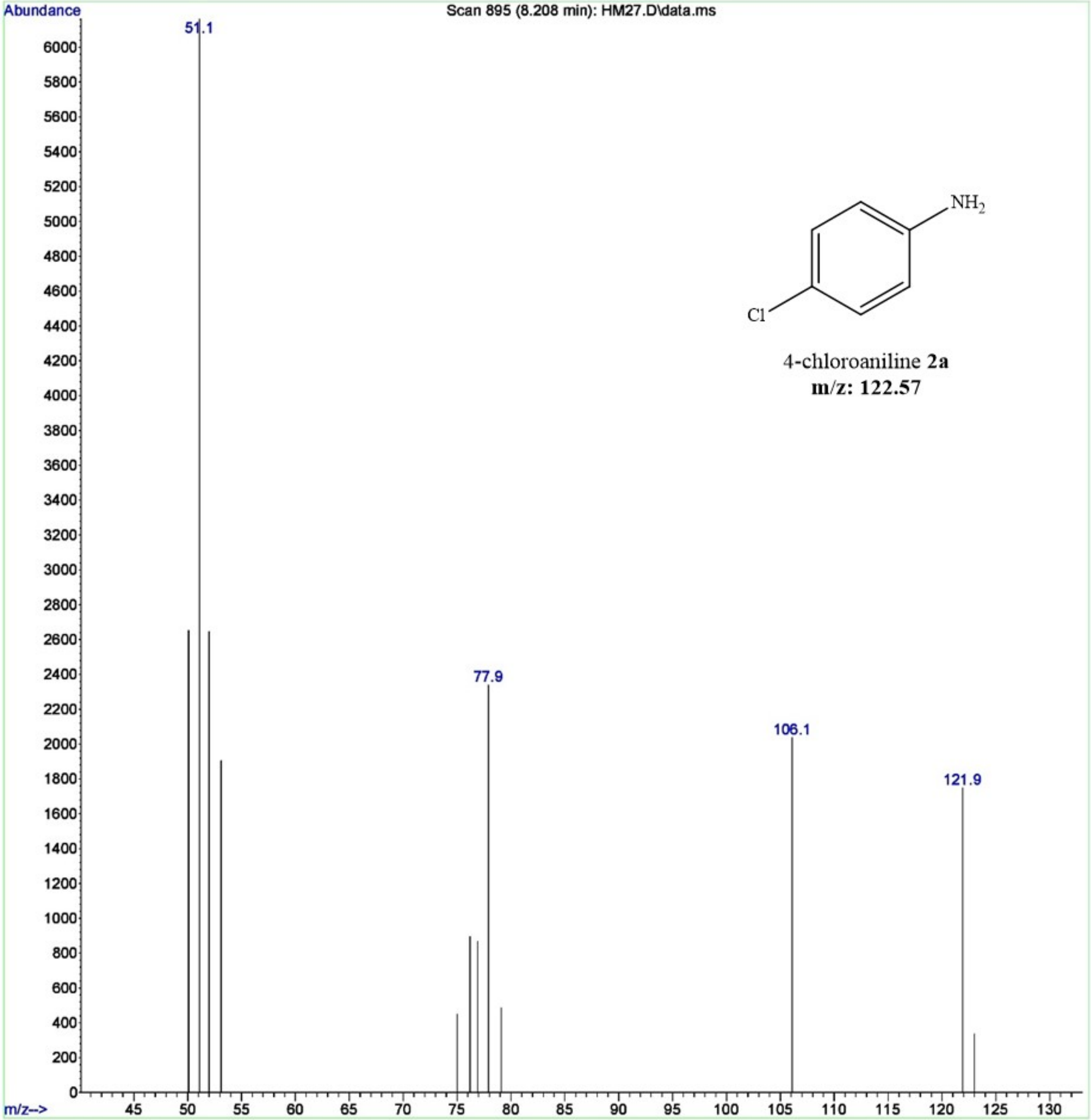

**4-bromoaniline**

Instrument : GCMSD
Sample Name: GNB-L
Misc Info :
Vial Number: 1

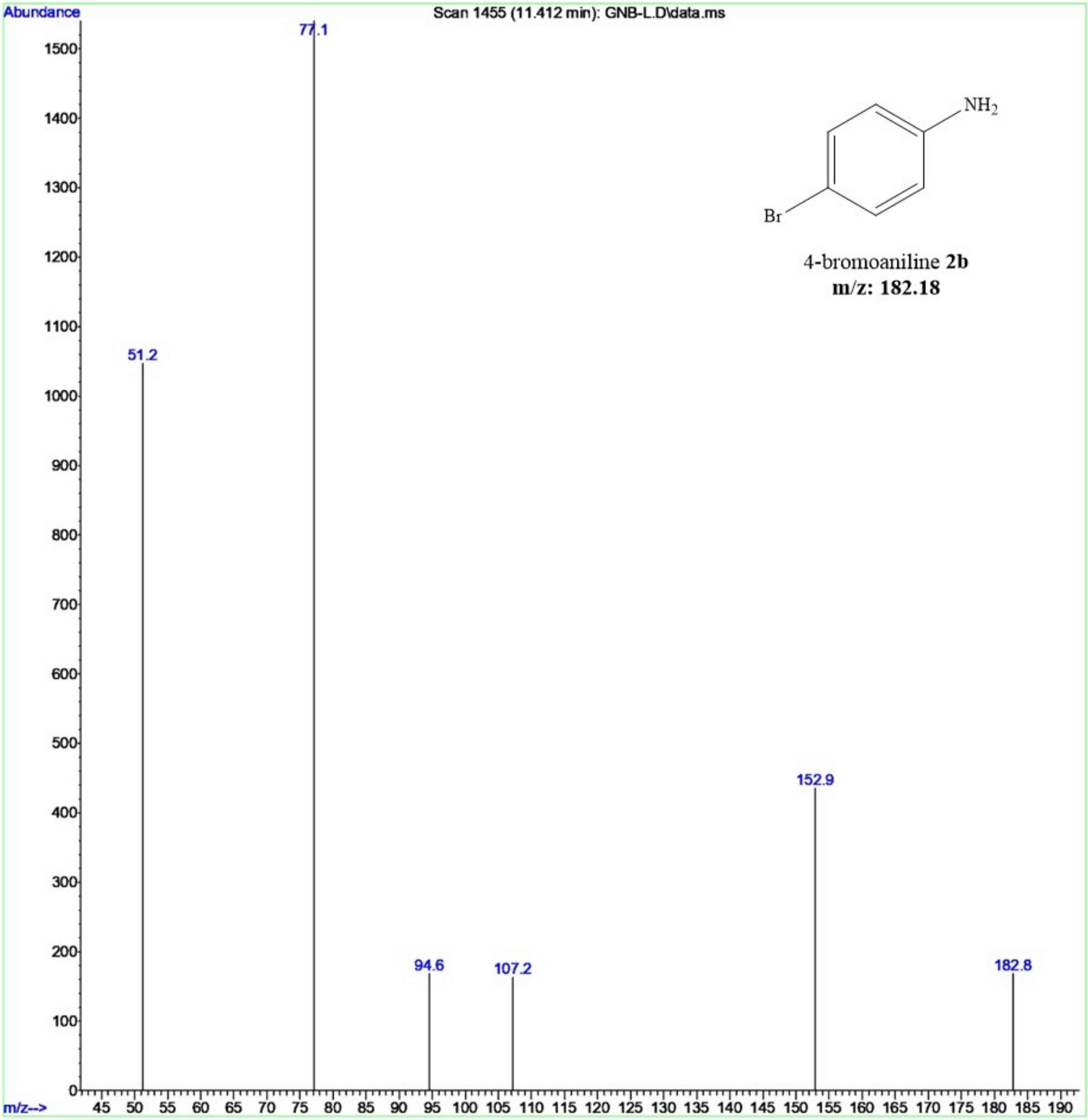

# 4-fluoroaniline

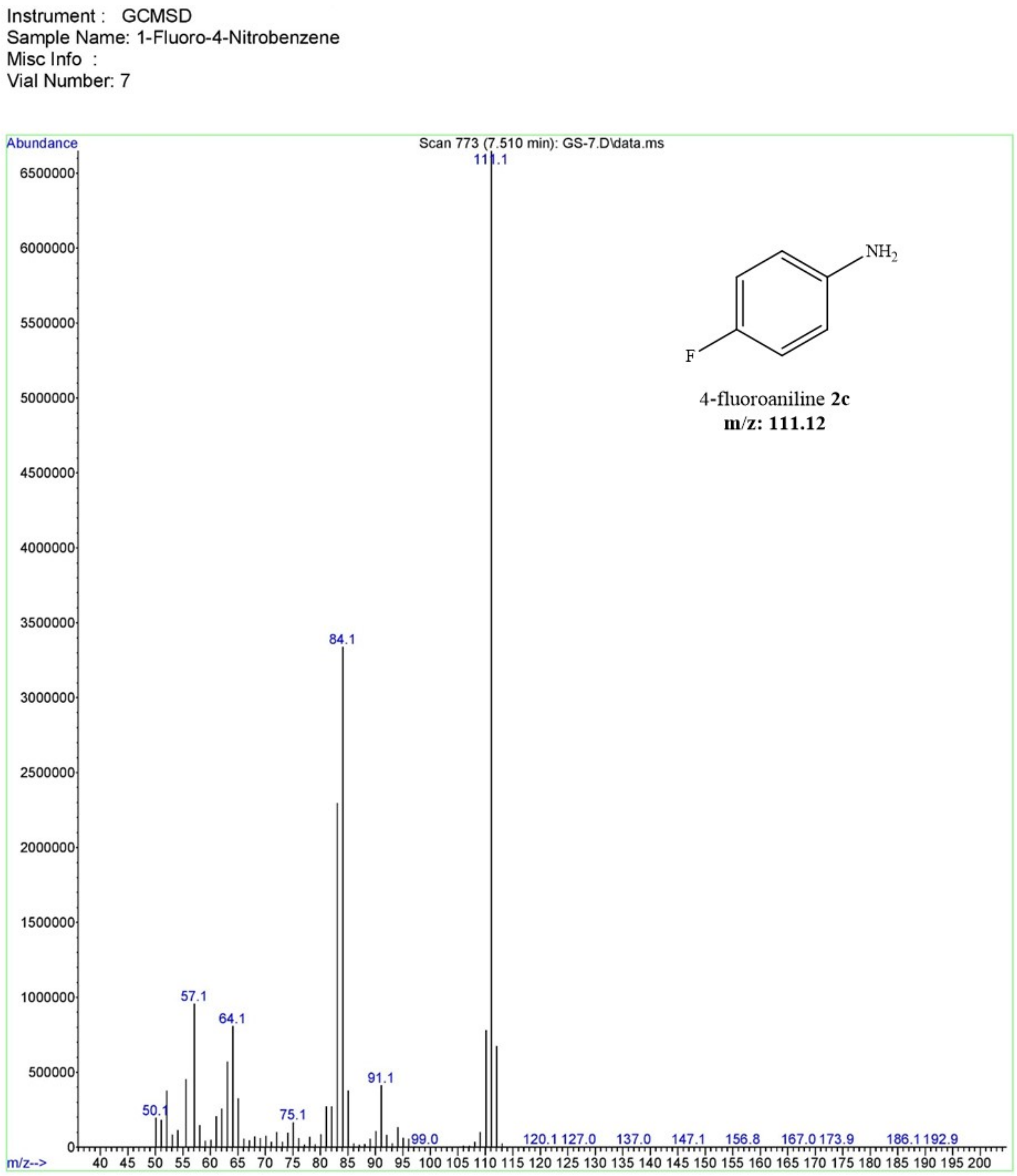

**3-chloro-4-nitroaniline**

Instrument : GCMSD
Sample Name: 1-Chloro-2,5-Dinitrobenzene
Misc Info :
Vial Number: 5

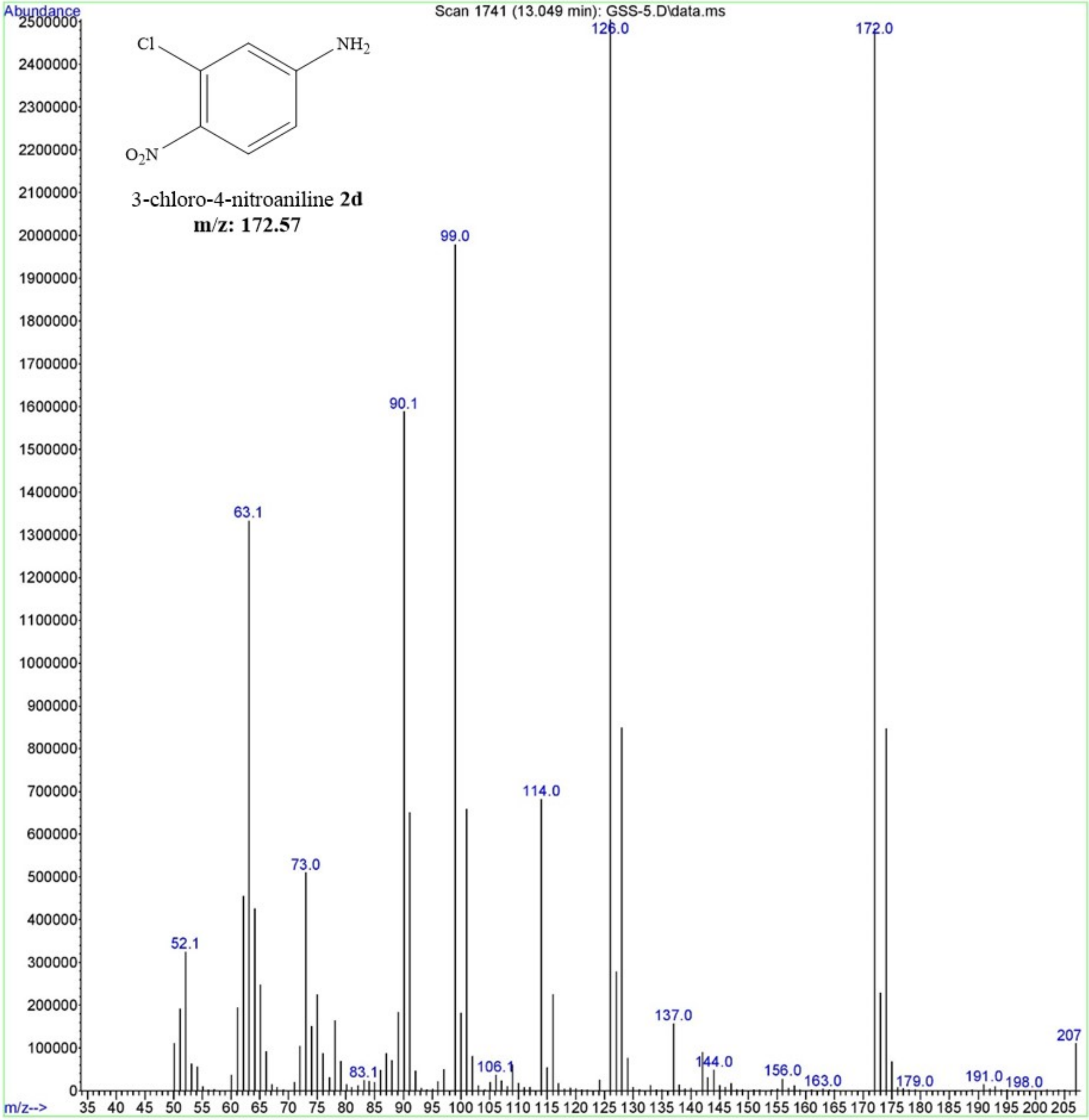

## 2-chloro-4-nitroaniline

Instrument : GCMSD
Sample Name: 1-Chloro-2,5-Dinitrobenzene
Misc Info :
Vial Number: 5

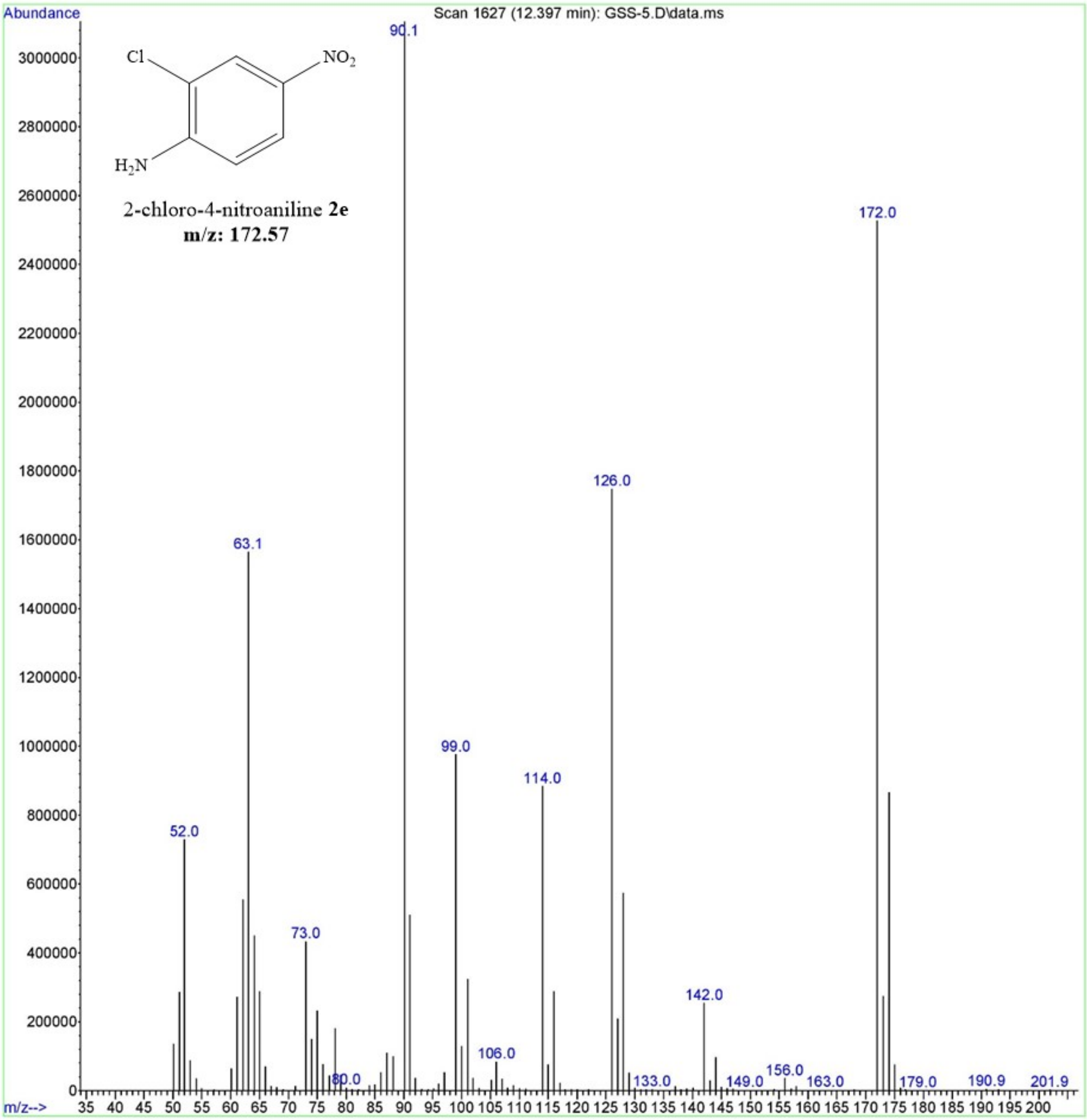

**2,5-dichloroaniline**

Instrument : GCMSD
Sample Name: 2,5-Dichloro-1-Nitrobenzene
Misc Info :
Vial Number: 3

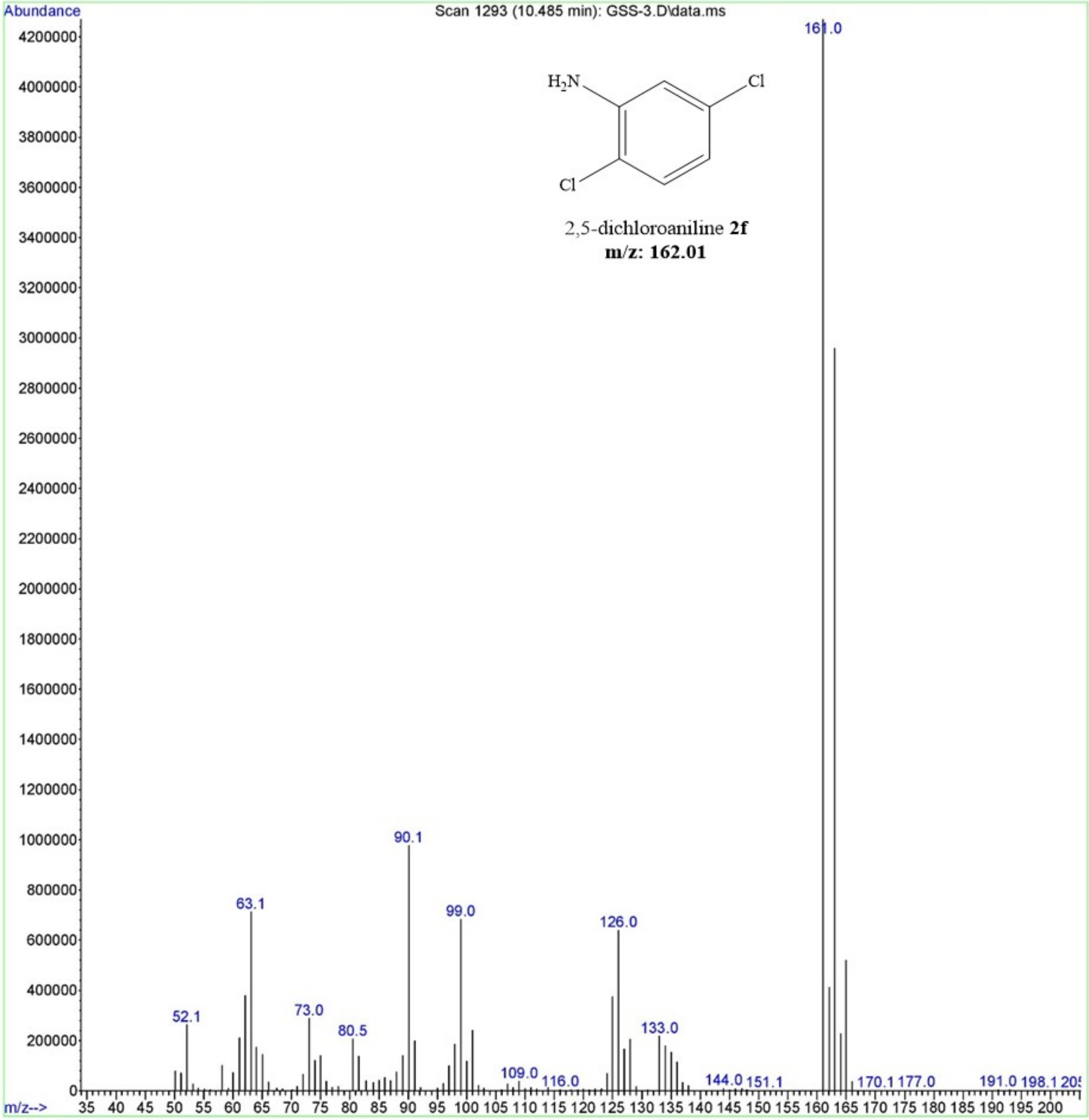

**4-aminobenzonitrile**

Instrument : GCMSD
Sample Name: 4-Nitrobenzonitrile
Misc Info :
Vial Number: 1

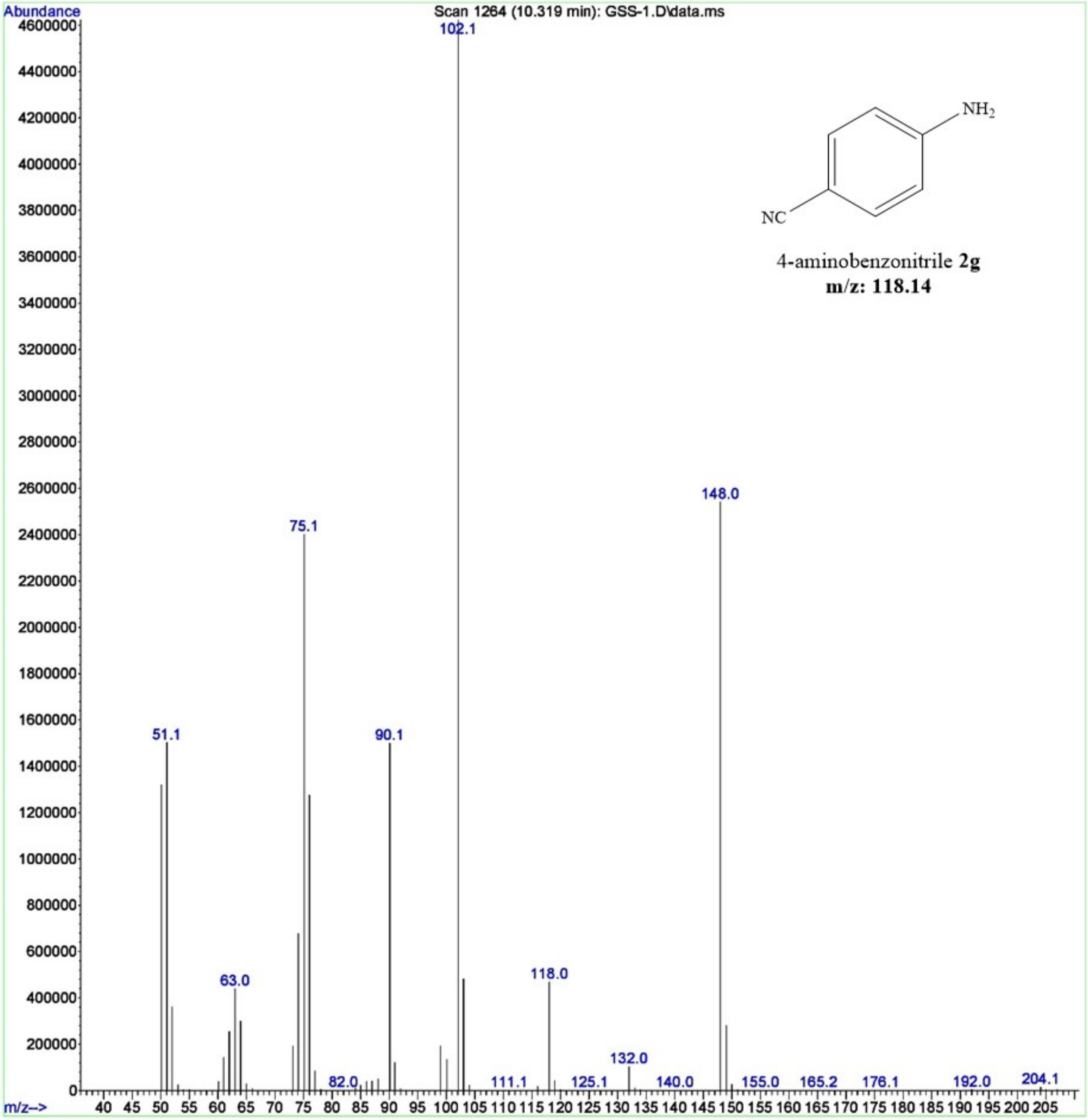

**4-methoxyaniline**

Instrument : GCMSD
Sample Name: 4-Nitroanisole
Misc Info :
Vial Number: 9

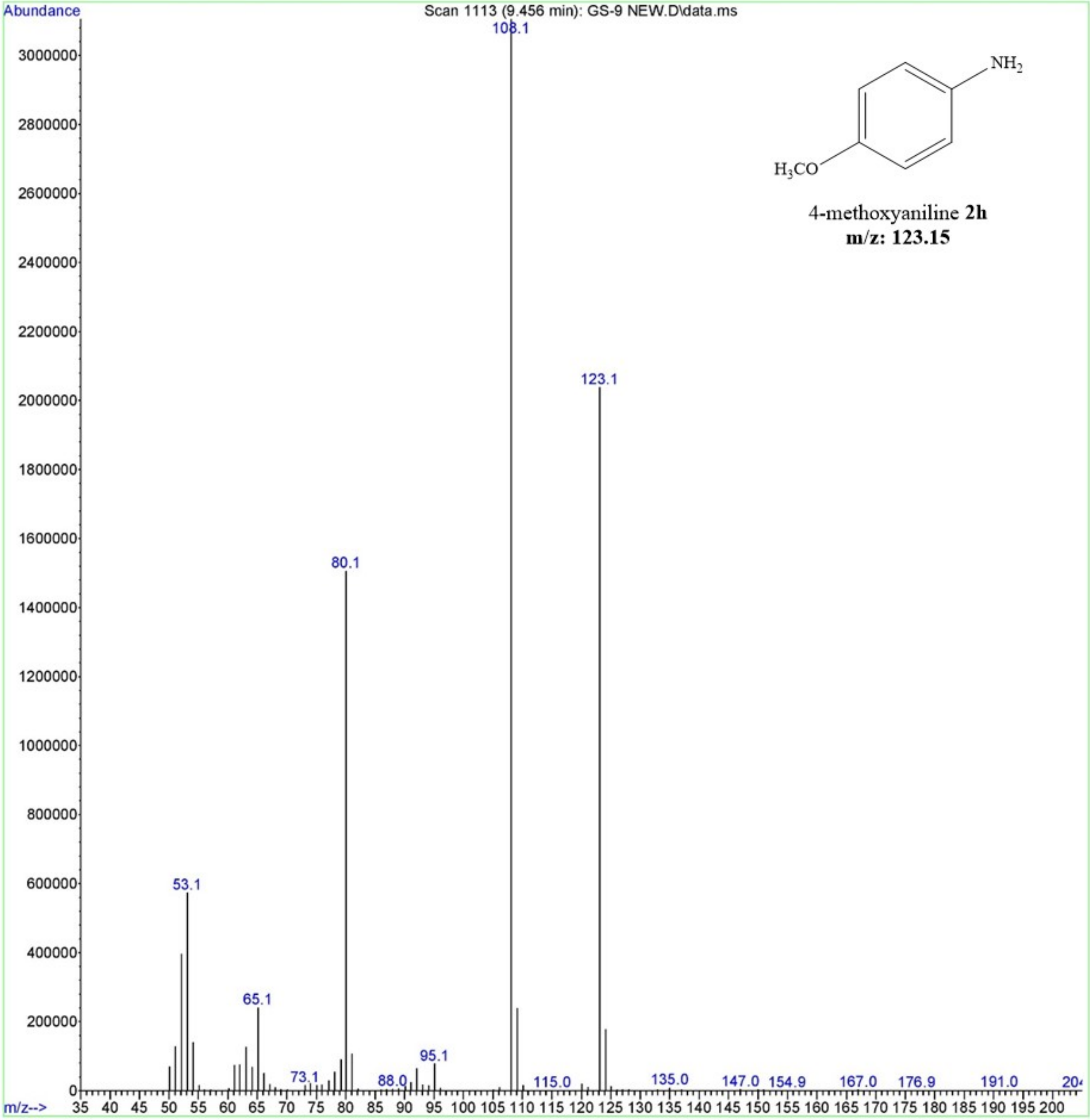

**4-methylaniline**

Instrument : GCMSD
Sample Name: 4-Nitrotoluene
Misc Info :
Vial Number: 4

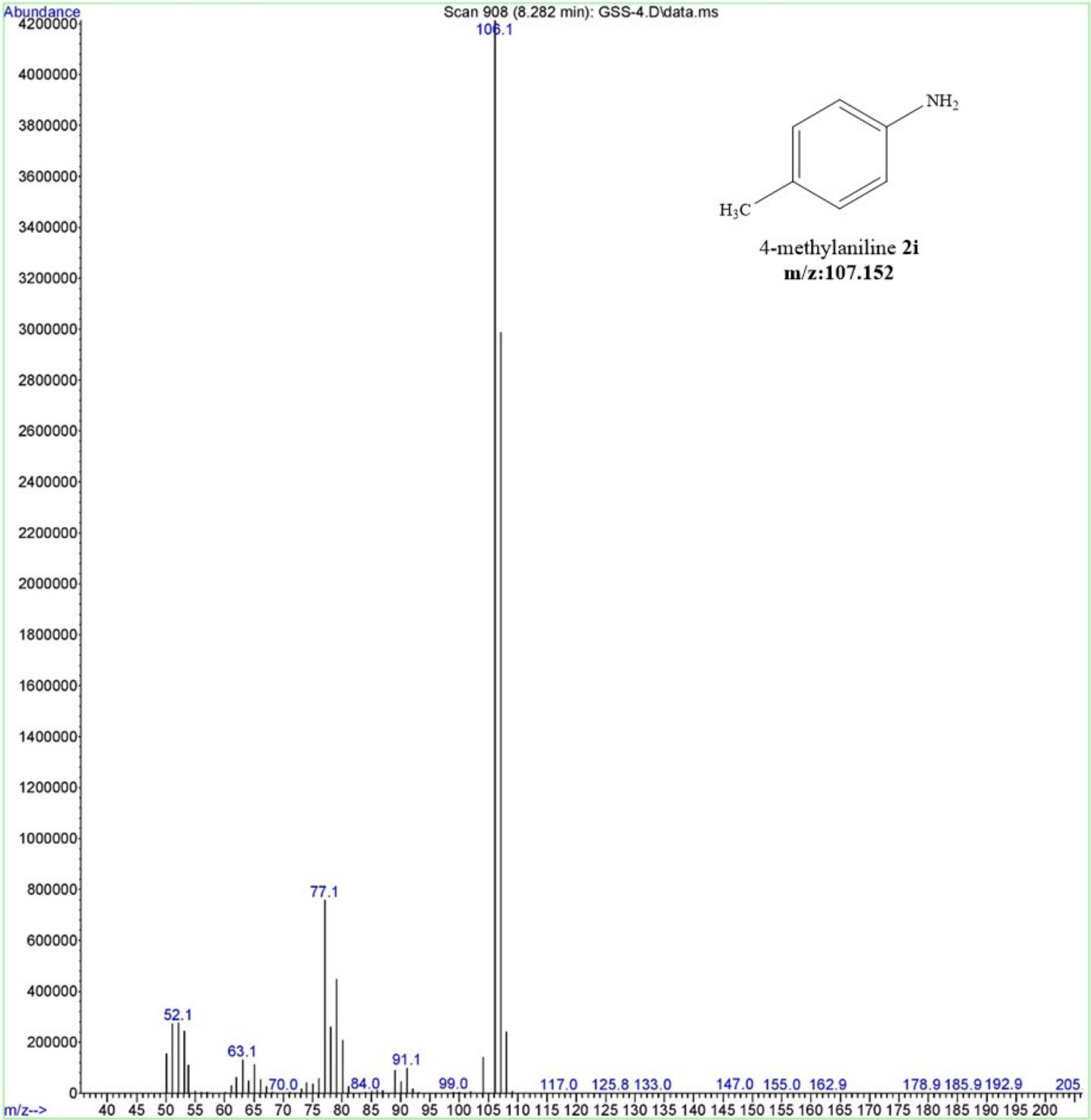

## 4-aminobenzaldehyde

Instrument : GCMSD
Sample Name: 4-Nitrobenzaldehyde
Misc Info :
Vial Number: 8

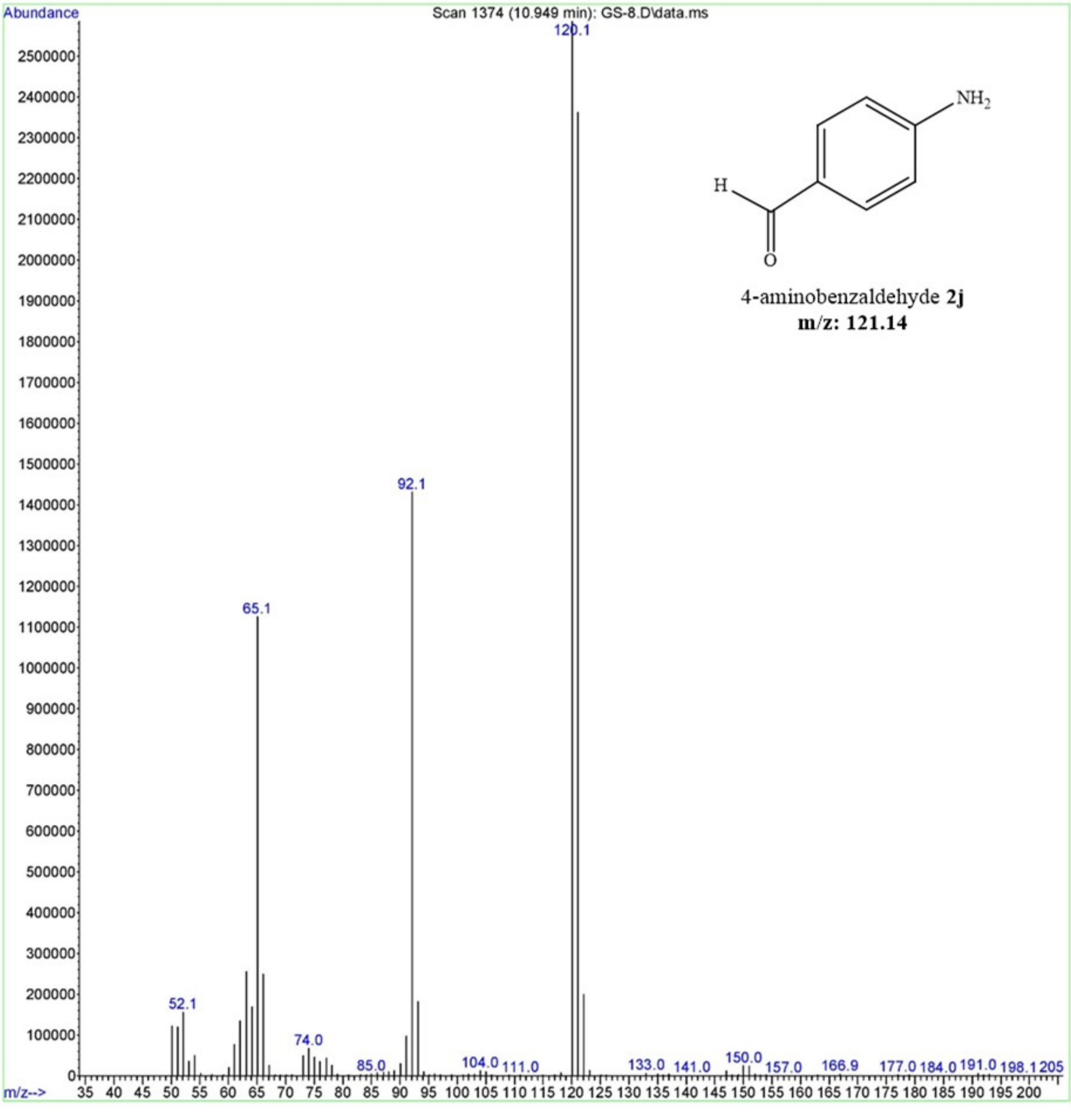

**4-aminoacetophenone**

Instrument : GCMSD
Sample Name: 4-Nitroacetophenon
Misc Info :
Vial Number: 2

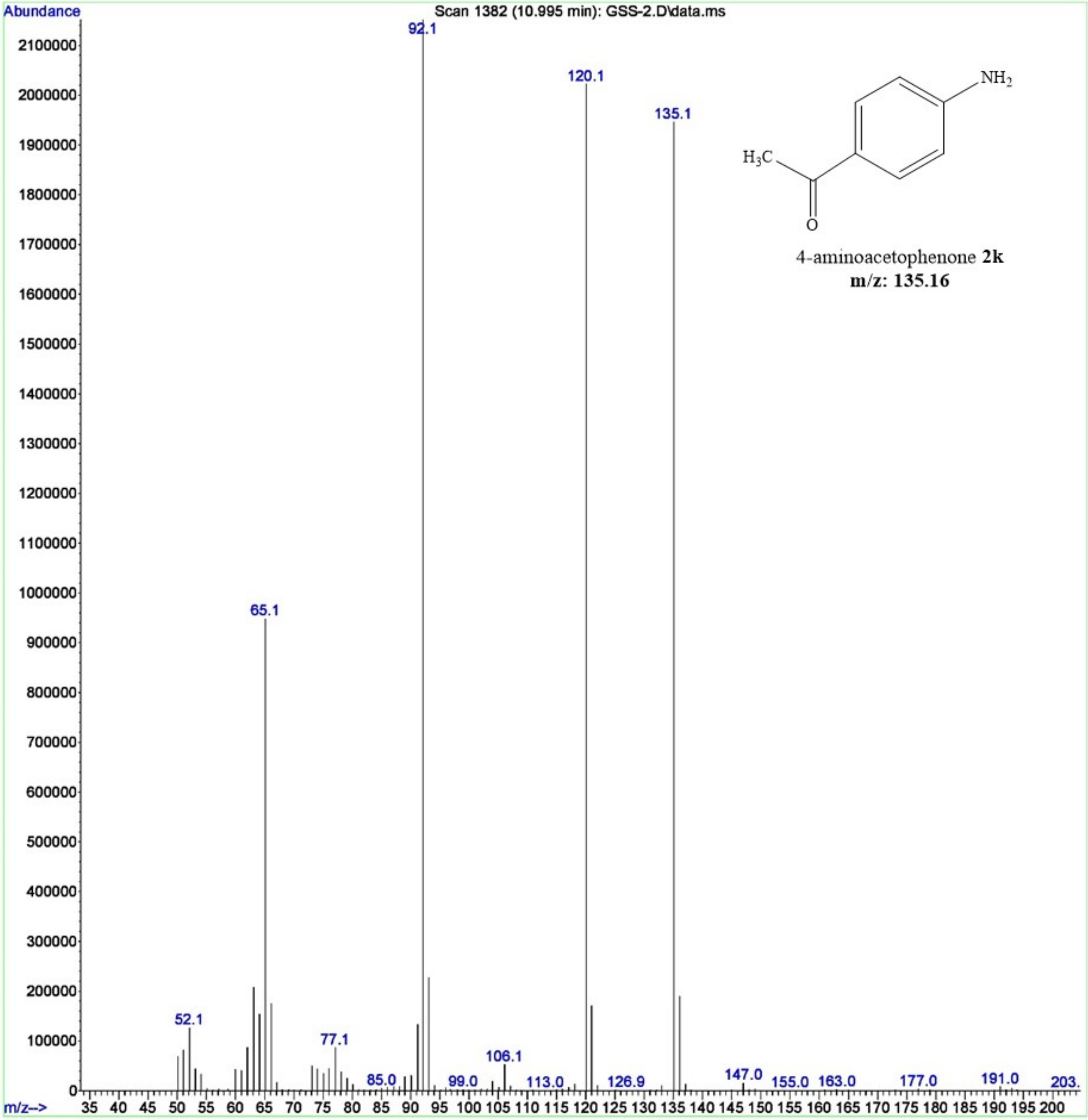

## 4-aminobenzoic acid

Instrument : GCMSD
Sample Name: 4-nitrobenzoic acid
Misc Info :
Vial Number: 6

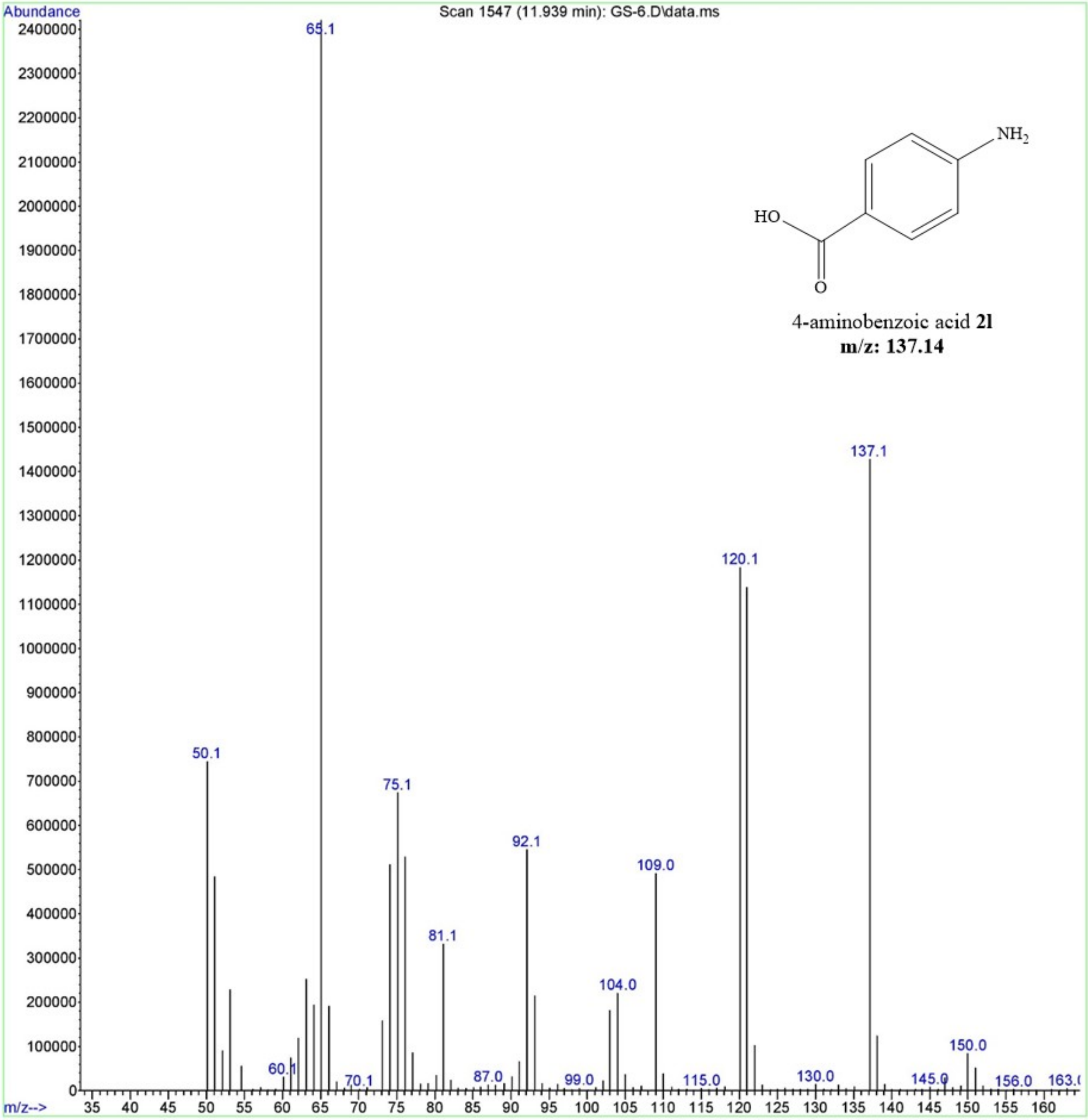

## Nitrile Hydration

## Nicotinamide

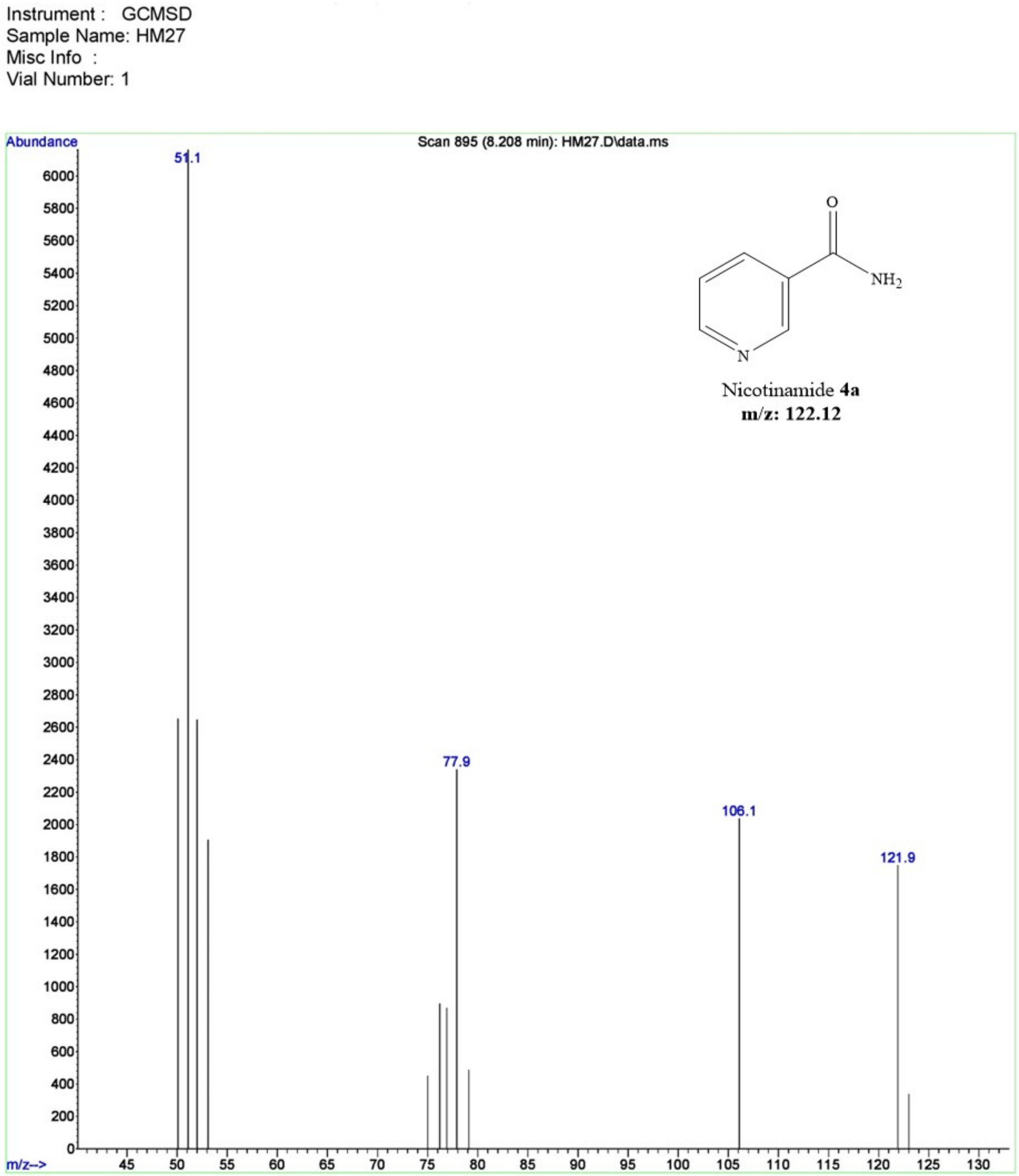

## Isonicotinamide

Instrument : GCMSD
Sample Name: GNB-F
Misc Info :
Vial Number: 1

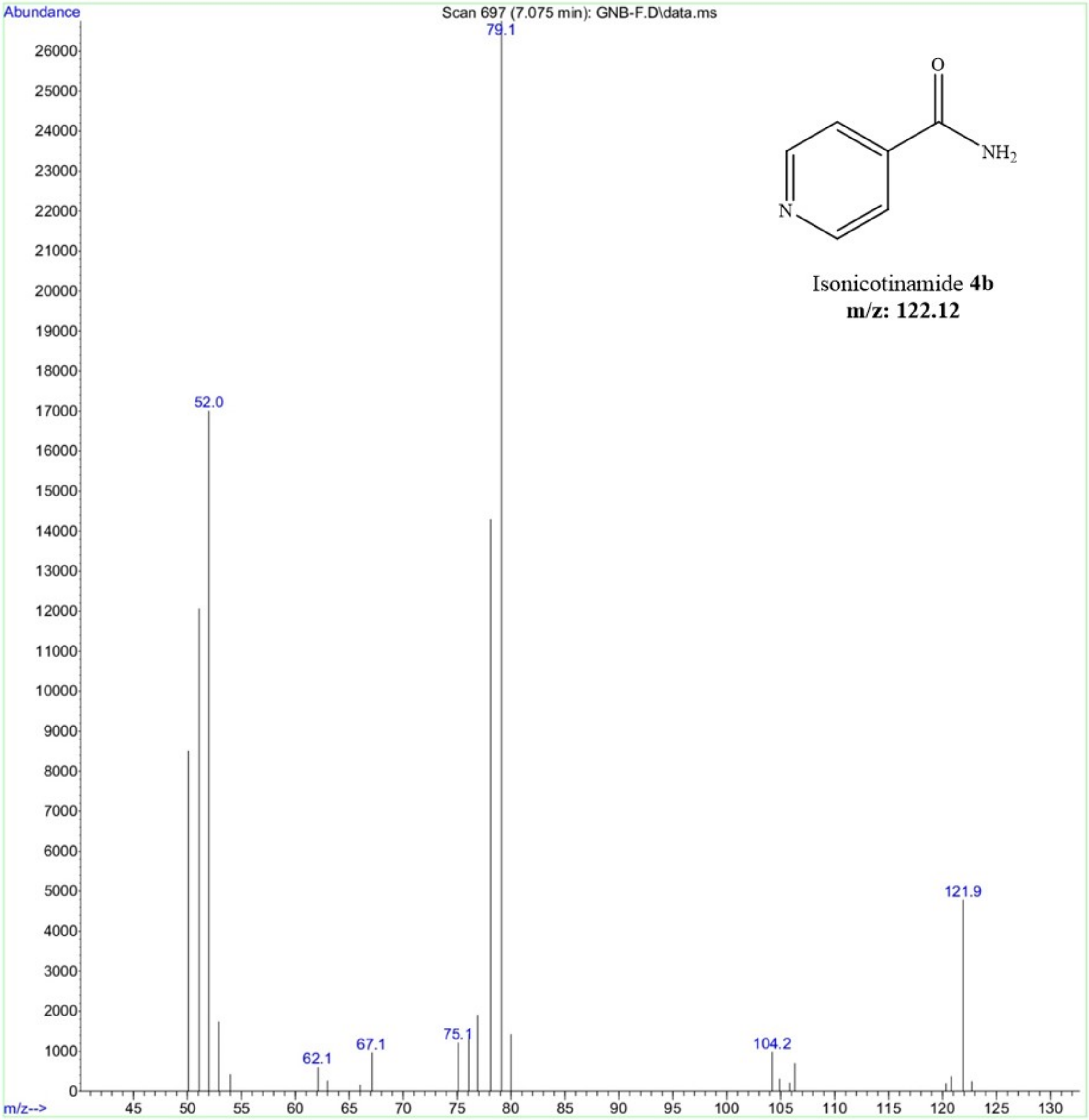

**Picolinamide**

Instrument : GCMSD
Sample Name: CB-1
Misc Info :
Vial Number: 1

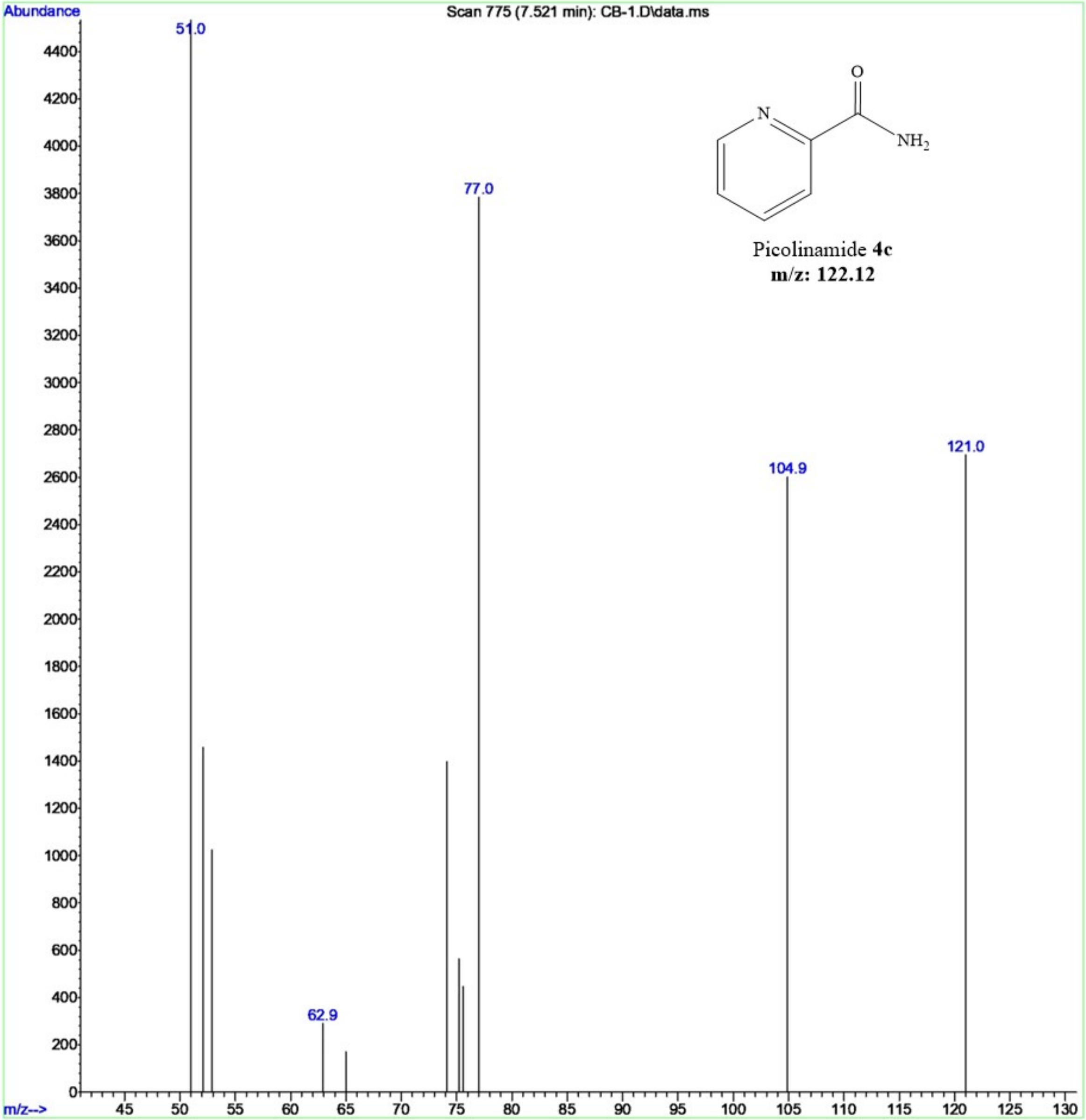

**4-fluorobenzamide**

Instrument : GCMSD
Sample Name: HM-28
Misc Info :
Vial Number: 1

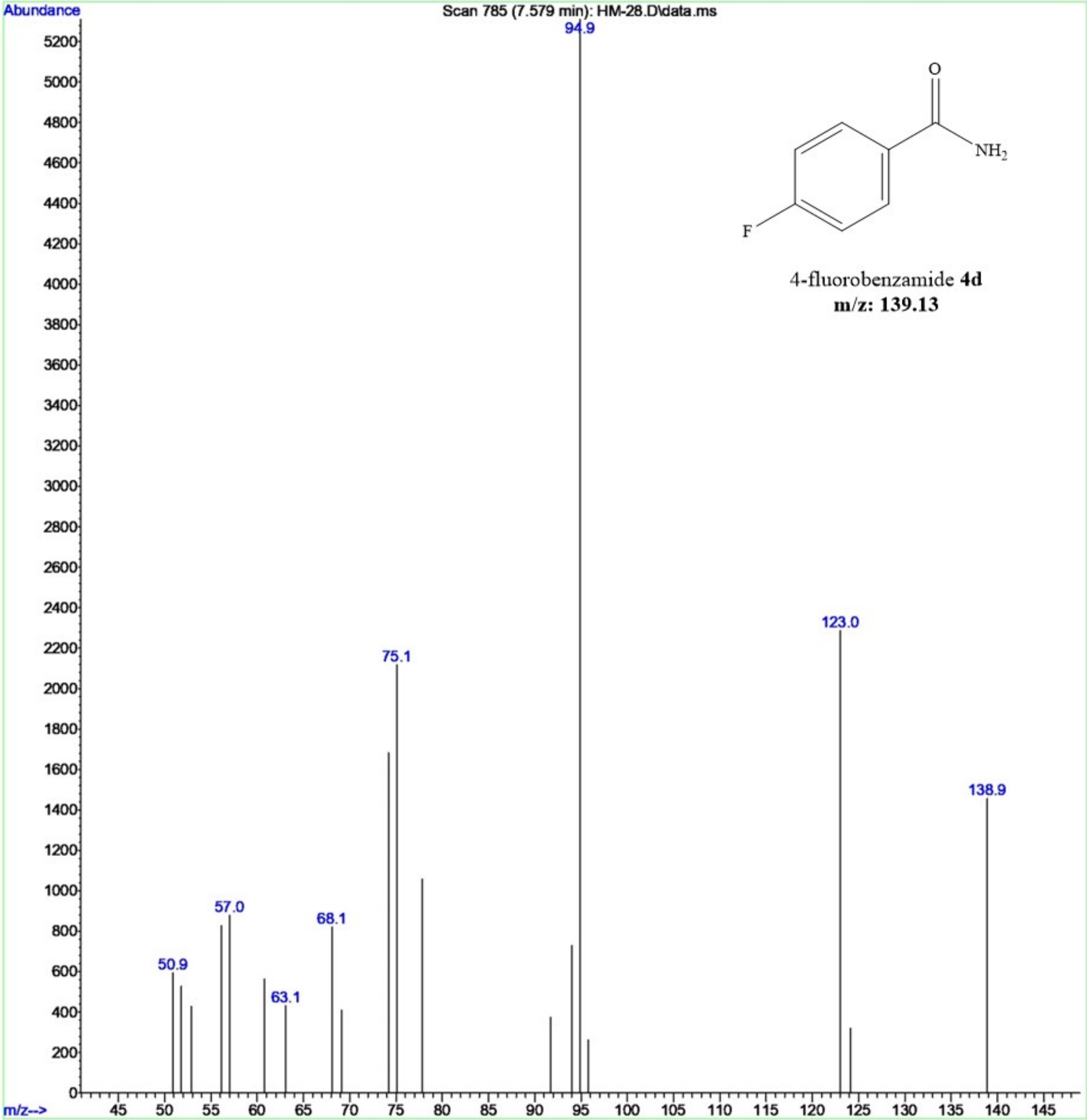

**4-chlorobenzamide**

Instrument : GCMSD
Sample Name: CC
Misc Info :
Vial Number: 1

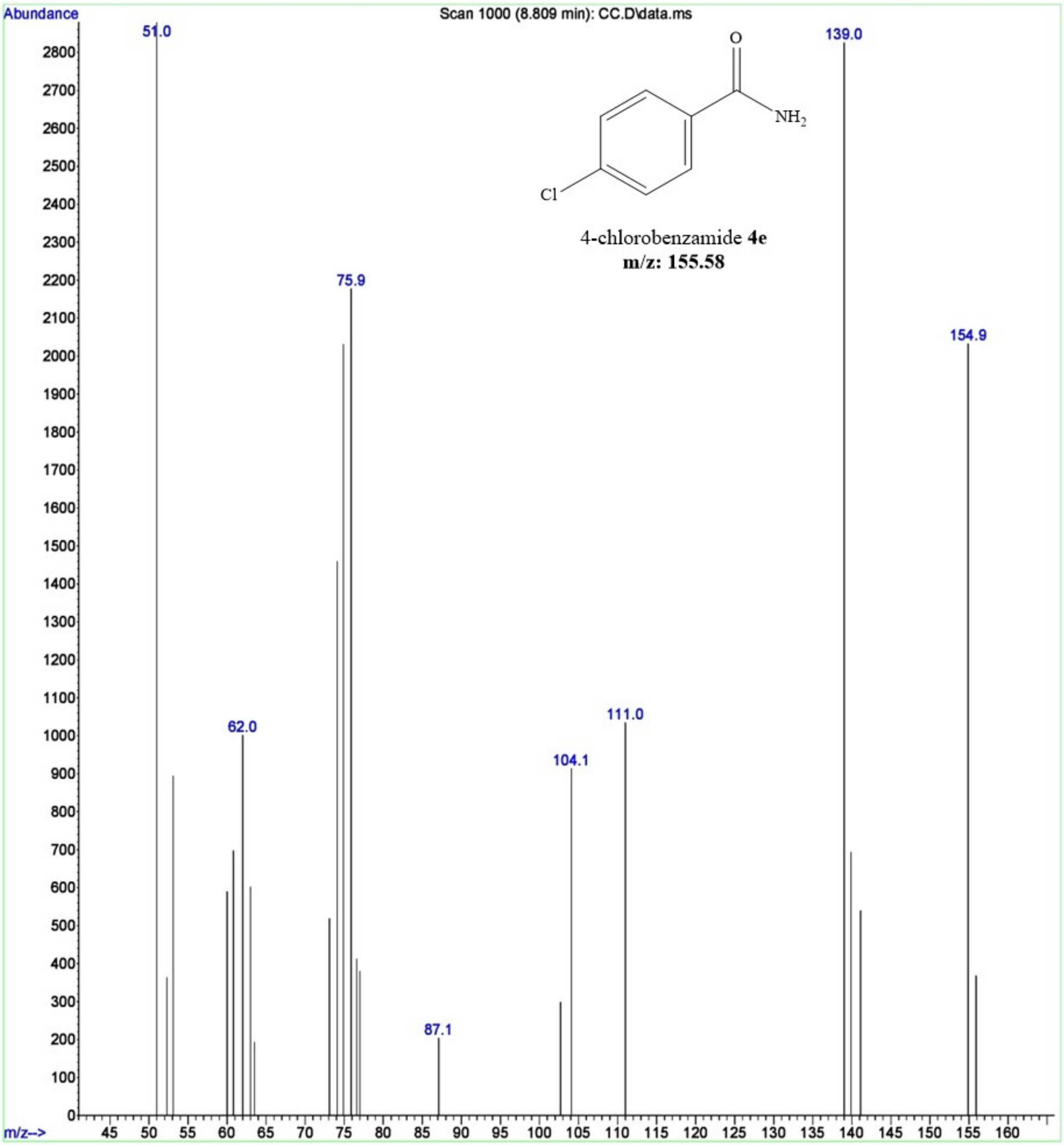

**4-bromobenzamide**

Instrument : GCMSD
Sample Name: CB
Misc Info :
Vial Number: 1

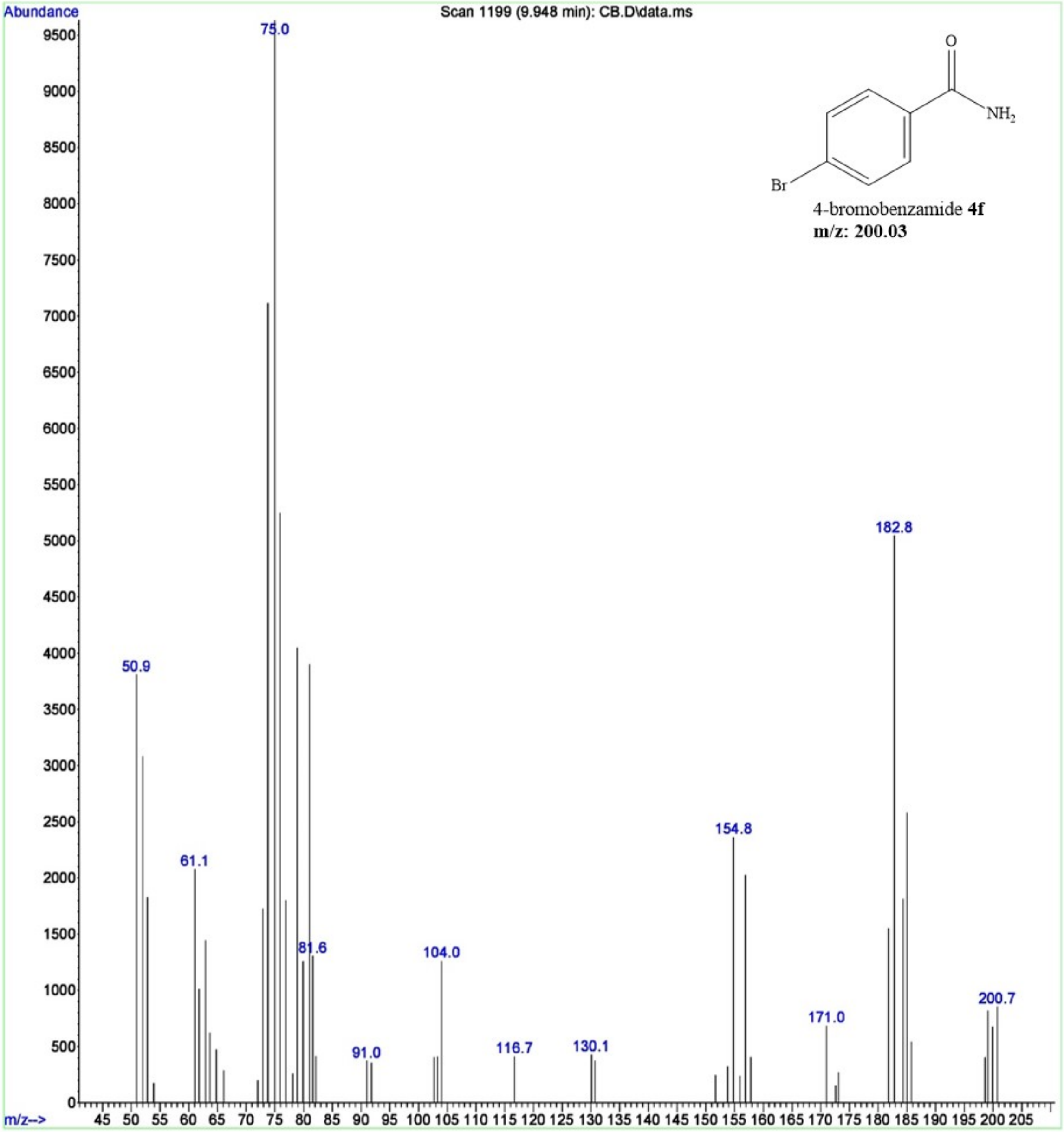

## 4-methylbenzamide

Instrument : GCMSD
Sample Name: 38
Misc Info :
Vial Number: 1

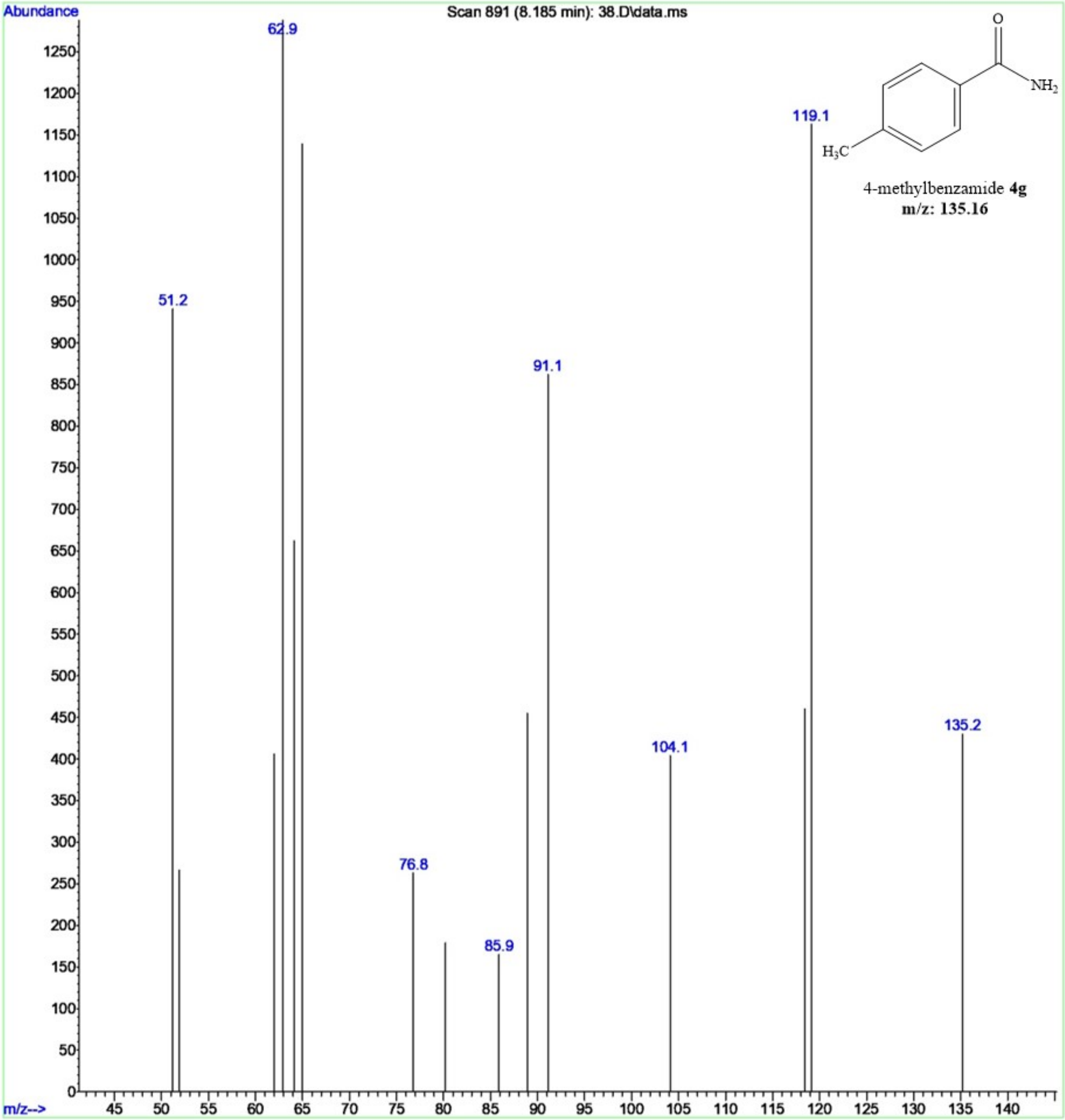

**Benzamide**

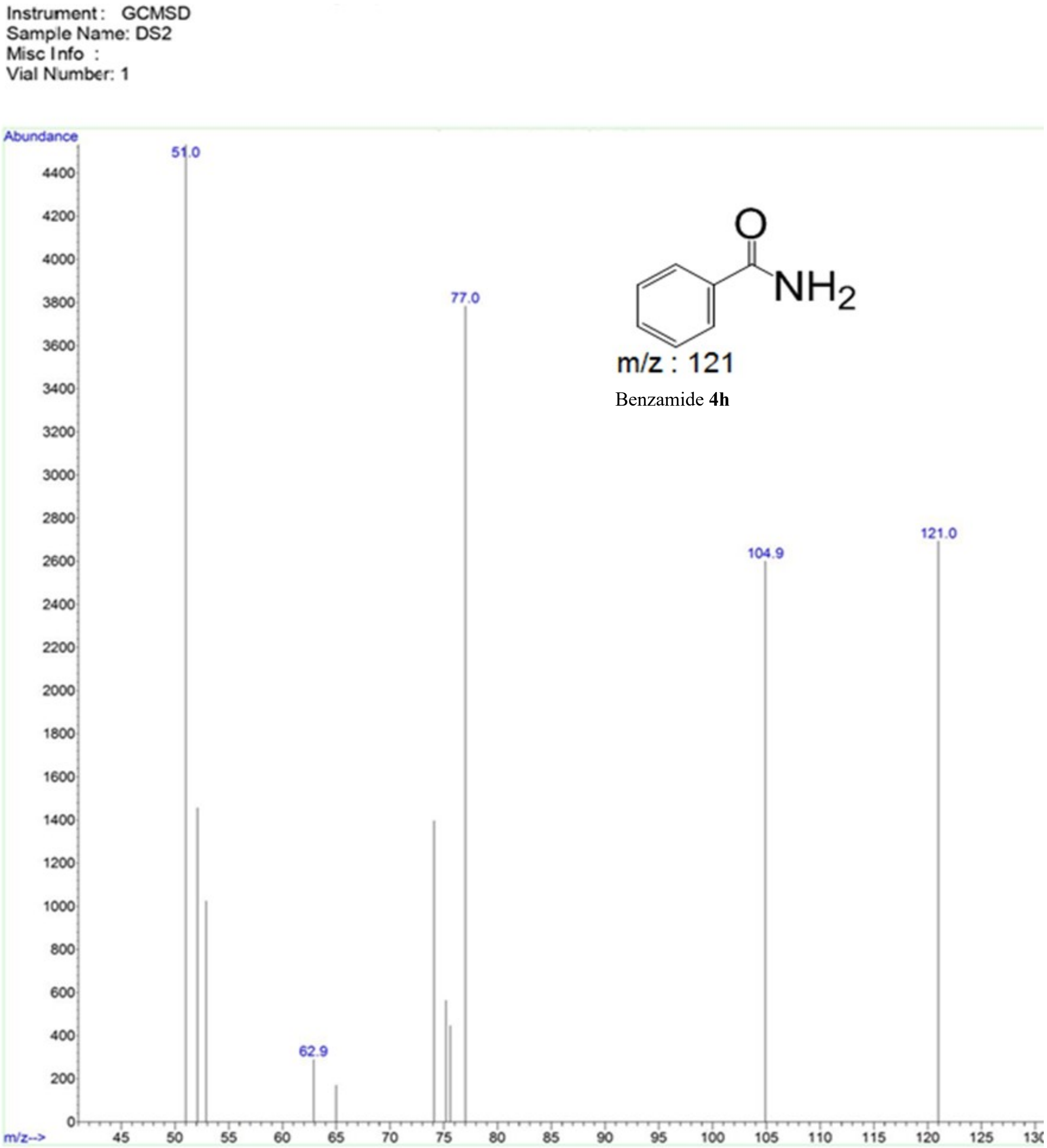

Benzamide **4h**

**4-methoxybenzamide**

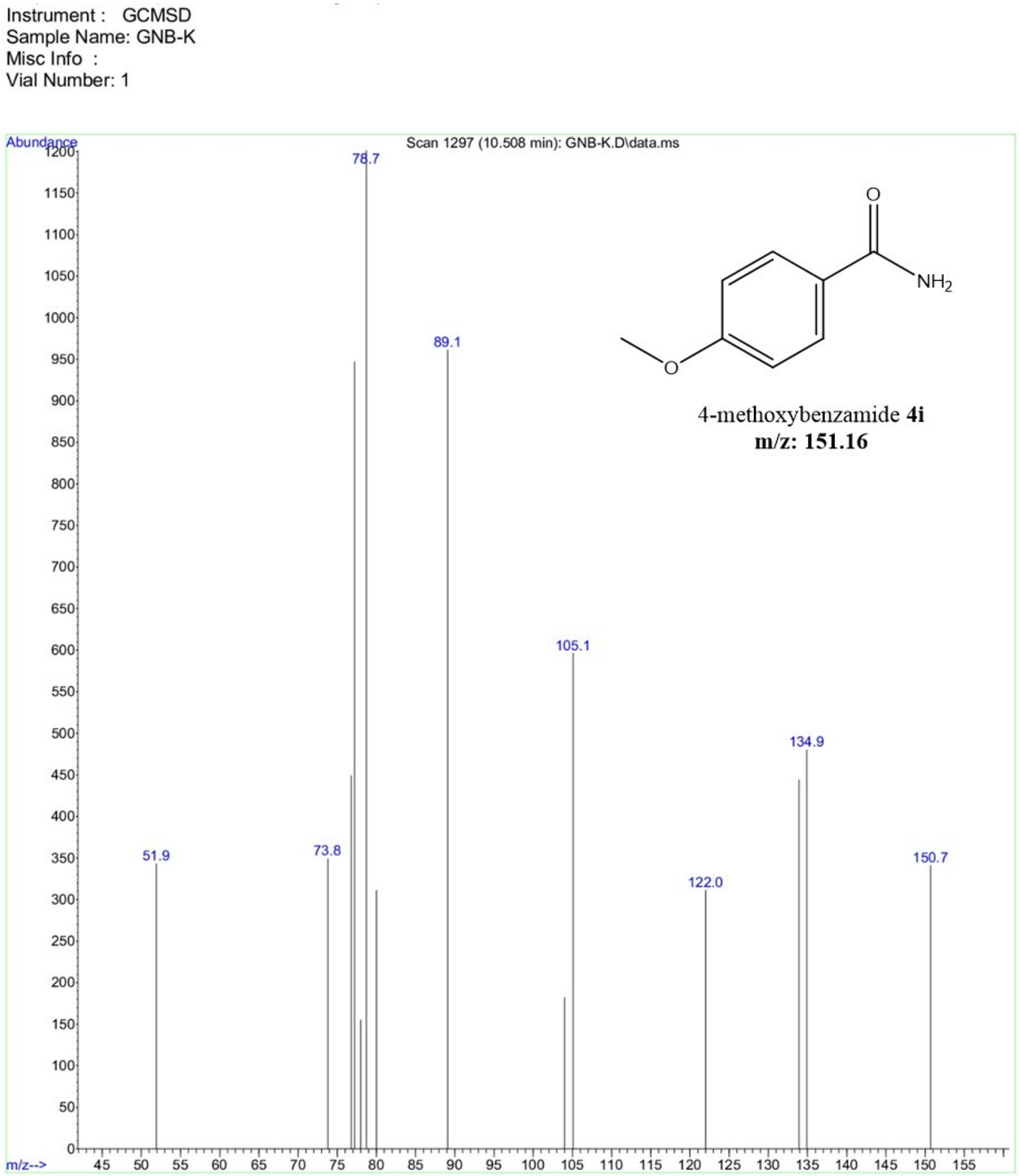

**4-nitrobenzamide**

Instrument : GCMSD
Sample Name: GNB-D
Misc Info :
Vial Number: 1

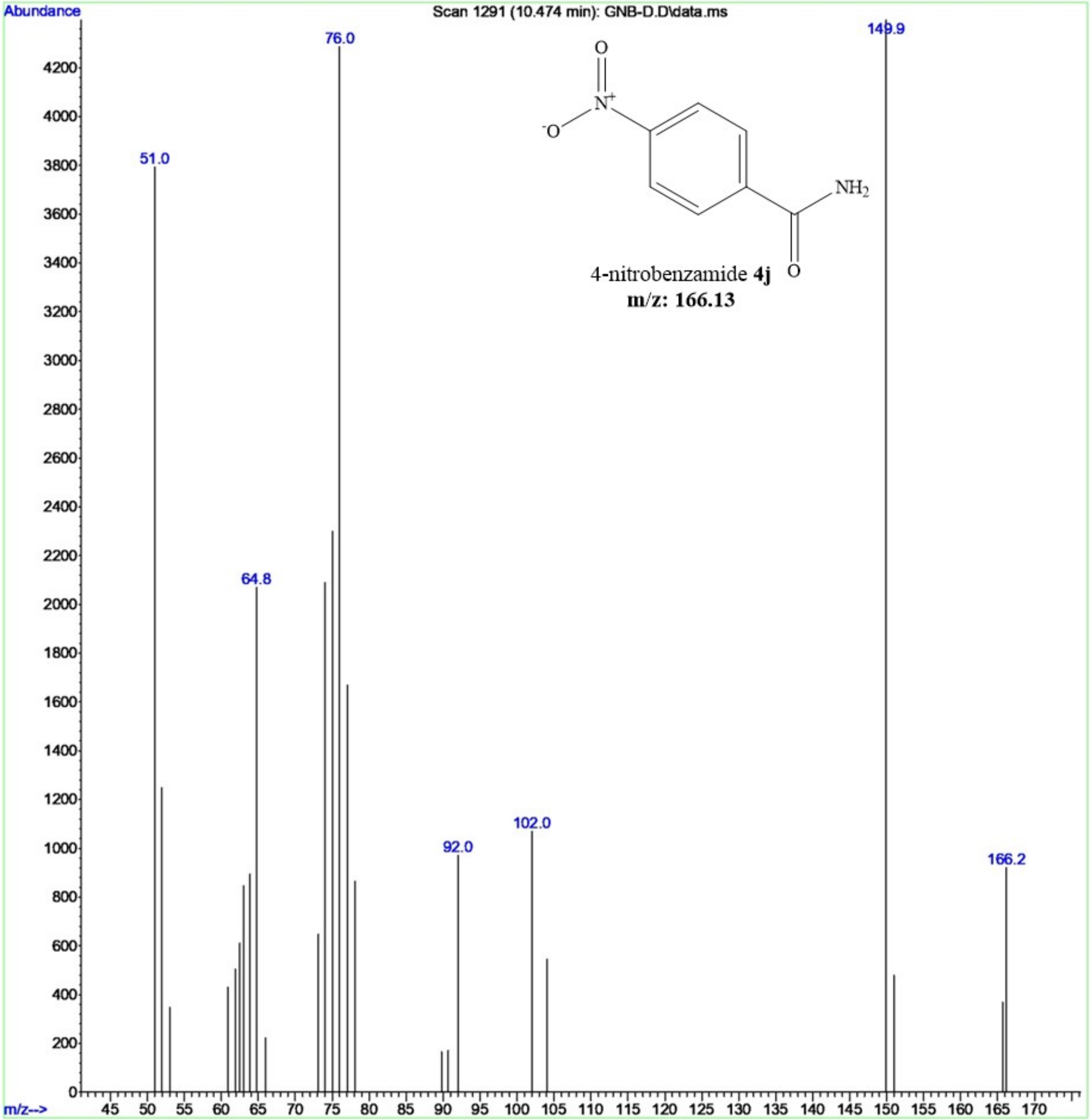

## 4-aminobenzamide

Instrument : GCMSD
Sample Name: GNB-K
Misc Info :
Vial Number: 1

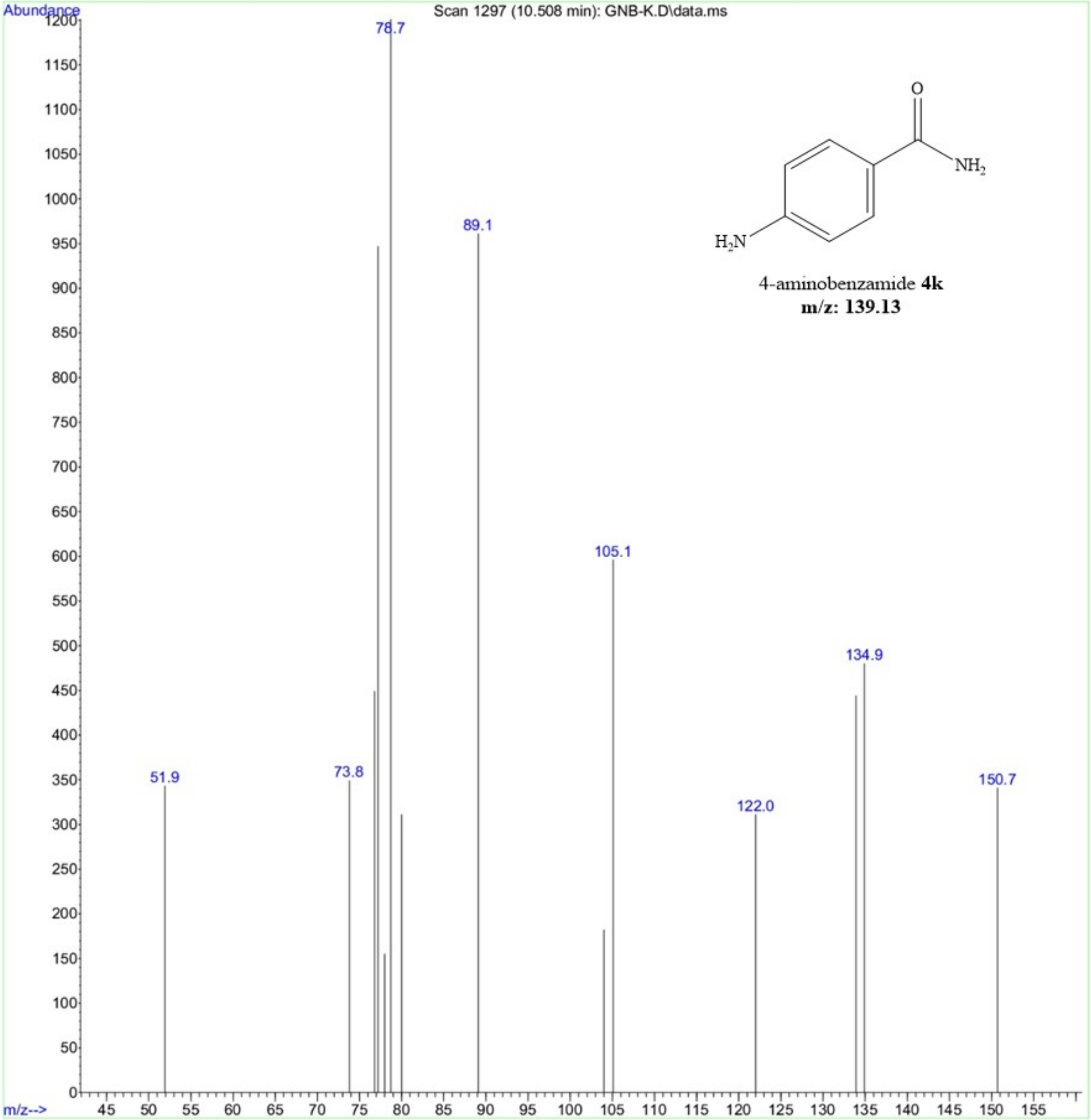

## 4-(bromomethyl)benzamide

Instrument : GCMSD
Sample Name: GNB-H
Misc Info :
Vial Number: 1

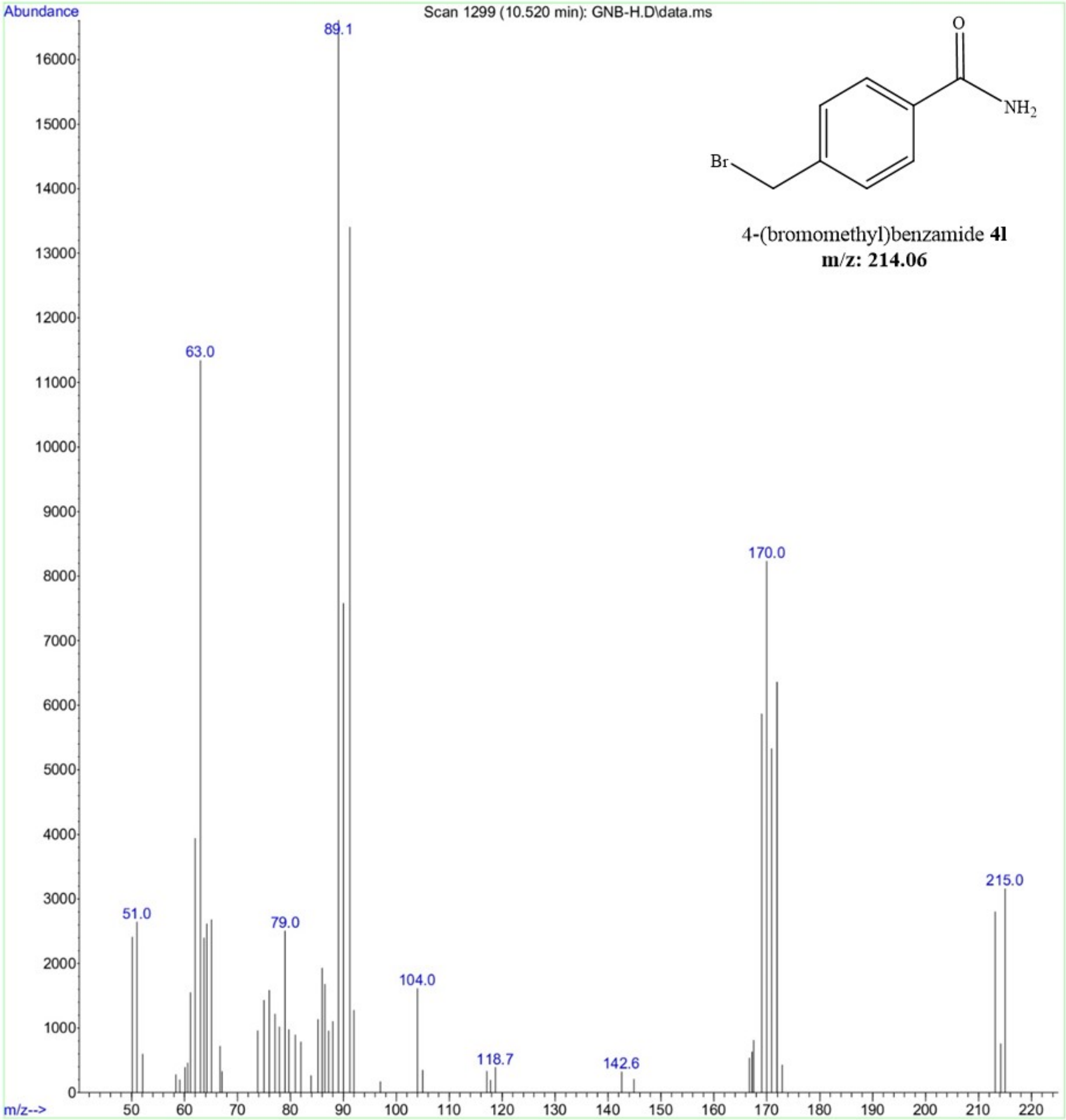

## 4-(cyanomethyl)benzamide

Instrument : GCMSD
Sample Name: GNB-D
Misc Info :
Vial Number: 1

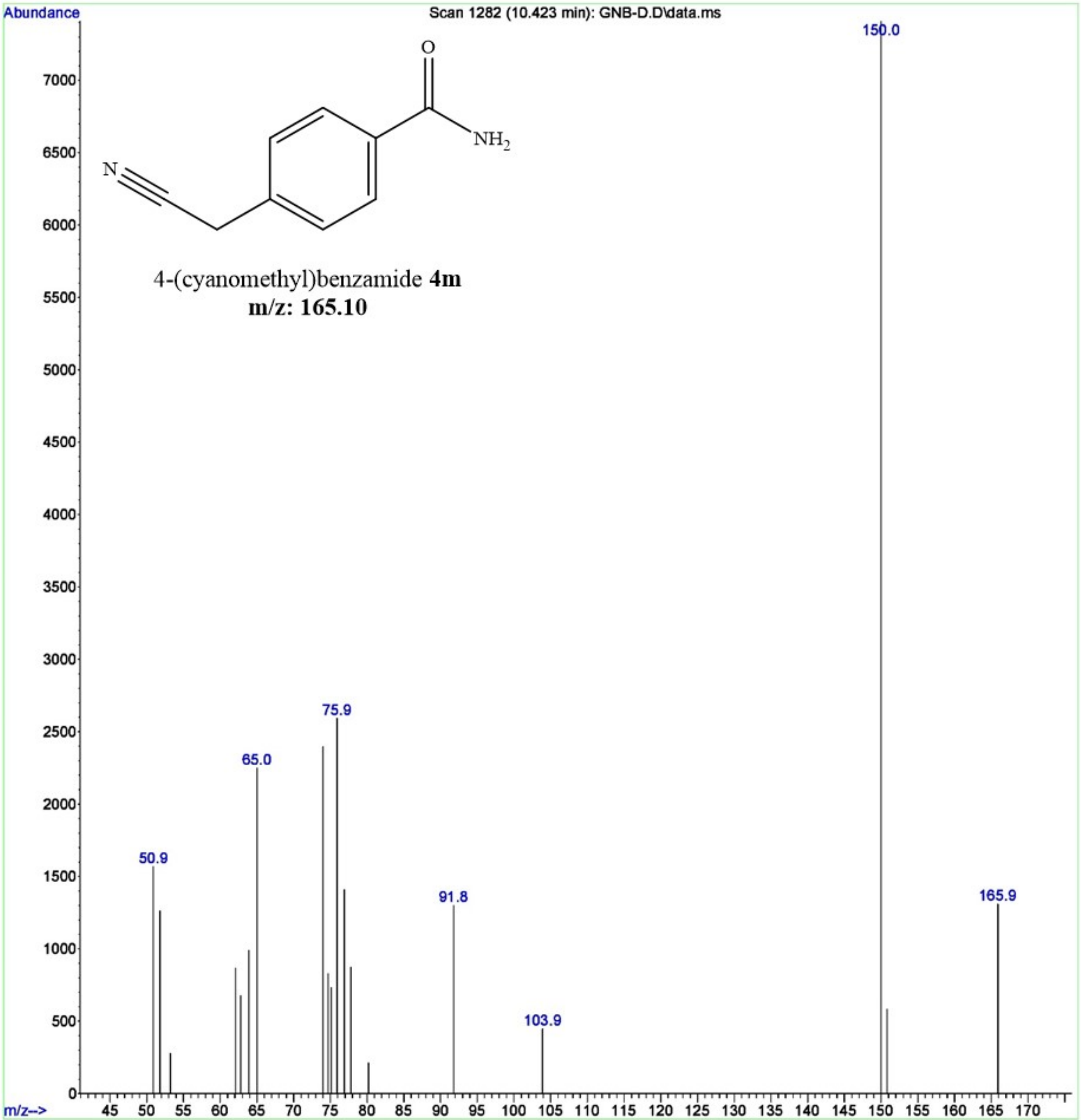

## Notes and references


1 Y. M. Jayawardena H. S. N., Liyanage Sajani H., Rathnayake K., Patel U., *Anal. Chem.*, 2021, **93**, 1889–1911.

2 A. Müller, T. Heinrich, S. Tougaard, W. S. M. Werner, M. Hronek, V. Kunz, J. Radnik, J. M. Stockmann, V. D. Hodoroaba, S. Benemann, N. Nirmalananthan-Budau, D. Geißler, K. Sparnacci and W. E. S. Unger, *J. Phys. Chem. C*, 2019, **123**, 29765–29775.
